# Identification of fibronectin 1 (FN1) and complement component 3 (C3) as immune infiltration-related biomarkers for diabetic nephropathy using integrated bioinformatic analysis

**DOI:** 10.1080/21655979.2021.1960766

**Published:** 2021-08-23

**Authors:** Yuejun Wang, Mingming Zhao, Yu Zhang

**Affiliations:** a Department of Nephrology, Zhejiang Aged Care Hospital, Hangzhou Normal University, Hangzhou, Zhejiang, China; b Department of Nephrology, Xiyuan Hospital, China Academy of Chinese Medical Sciences, Beijing, China

**Keywords:** Biological markers, bioinformatics, diabetic nephropathy, macrophages, immune cell infiltration

## Abstract

Immune cell infiltration (ICI) plays a pivotal role in the development of diabetic nephropathy (DN). Evidence suggests that immune-related genes play an important role in the initiation of inflammation and the recruitment of immune cells. However, the underlying mechanisms and immune-related biomarkers in DN have not been elucidated. Therefore, this study aimed to explore immune-related biomarkers in DN and the underlying mechanisms using bioinformatic approaches. In this study, four DN glomerular datasets were downloaded, merged, and divided into training and test cohorts. First, we identified 55 differentially expressed immune-related genes; their biological functions were mainly enriched in leukocyte chemotaxis and neutrophil migration. The CIBERSORT algorithm was then used to evaluate the infiltrated immune cells; macrophages M1/M2, T cells CD8, and resting mast cells were strongly associated with DN. The ICI-related gene modules as well as 25 candidate hub genes were identified to construct a protein-protein interactive network and conduct molecular complex detection using the GOSemSim algorithm. Consequently, FN1, C3, and VEGFC were identified as immune-related biomarkers in DN, and a related transcription factor–miRNA–target network was constructed. Receiver operating characteristic curve analysis was estimated in the test cohort; FN1 and C3 had large area under the curve values (0.837 and 0.824, respectively). Clinical validation showed that FN1 and C3 were negatively related to the glomerular filtration rate in patients with DN. Six potential therapeutic small molecule compounds, such as calyculin, phenamil, and clofazimine, were discovered in the connectivity map. In conclusion, FN1 and C3 are immune-related biomarkers of DN.

## Introduction

The incidence of diabetes has increased rapidly in recent years and has emerged as a major cause of chronic kidney disease worldwide. As of 2015, approximately 415 million people were living with diabetes worldwide and this is expected to increase to 693 million by 2045 [1]. Approximately 40% of these patients develop end-stage renal disease and require renal replacement therapy, such as peritoneal dialysis, hemodialysis, and kidney transplantation [2]. Current treatment strategies rely on renin-angiotensin-aldosterone system (RAAS) blockers and sodium glucose co-transporter 2 (SGLT2) inhibitors [3]. However, the therapeutic effect of these drugs on diabetic nephropathy (DN) is either by reducing glomerular intracapsular pressure or by decreasing hyperglycemia, but not from specific and precise targets of DN. In addition, not all patients benefit from these drugs because of the genetic heterogeneity and complexity of the disease [4]. Hence, it is imperative to identify new targets to enhance the efficacy of treatment of DN.

Traditionally, metabolic and hemodynamic factors have been the major causes of DN. However, increasing evidence points to the role of inflammation and immune cell infiltration in its development [5]. Compared with healthy controls, inflammatory cytokines such as intracellular adhesion molecule (ICAM)-1, tumor necrosis factor (TNF)-α, interleukin (IL)-1, and IL-6 are found to be increased in serum or peripheral blood cells in patients with DN [6]. Macrophages, neutrophils, and mast cells are heavily infiltrated and functionally active in the kidney and are important drivers of the inflammatory response and fibrosis in the diabetic kidney [7]. Therefore, exploring the immune mechanisms of DN and identifying new targets for immunotherapy is of great value.

Immunological mechanisms play a significant role in the development and progression of DN, with recruitment and activation of innate immune cells and the development of proinflammatory molecules [8]. The expression of some immune and inflammatory genes is upregulated in renal cells of animal models of diabetes as well as in patients with diabetes [9]. These genes play an important role in the initiation of inflammation and the recruitment of immune cells. Toll-like receptors (TLR)2 and TLR4 are highly expressed in tubular epithelial cells, endothelial cells, podocytes, and mesangial cells of patients suffering from diabetic injury in the kidney [10]. Elevated TLR4 levels in kidney samples of patients with diabetes are positively correlated with the infiltration of macrophages and negatively correlated with the glomerular filtration rate [11]. In diabetic patients, chemokine monocyte chemoattractant protein 1 (MCP1) is upregulated in the glomerular and renal tubular epithelium [12,13]. MCP1 is responsible for the migration of monocytes through the endothelium after adhesion and is a major factor influencing macrophage accumulation in renal disease patients and in animal models of renal damage [14]. With the rapid increase in high-throughput data, bioinformatic approaches have been applied to identify immune-related biomarkers in hypertension [15] and lung adenocarcinoma [16]. However, limited evidence is based on low-throughput experimental verification, and the study of immune genes in diabetic nephropathy through high-throughput data mining is still lacking.

With the development of bioinformatic technology, it has been gradually realized that human diseases are not caused by a single molecular defect but are driven by complex interactions between various molecules. The complexity of these interactions encompasses different types of information, ranging from cell-molecular level protein-protein interactions to related studies of gene expression and regulation, metabolic and disease pathways, and drug-disease relationships [17]. As a rapidly developing new field, network medicine combines molecular biology and network science and is expected to reveal the causes of human diseases and radically change their diagnosis and treatment [18]. Network medicine-based algorithms, such as protein-protein interaction (PPI) [19], switch genes miner (SWIM) [20] and weighted correlation network analysis (WGCNA) [21], have also been successfully used to investigate the mechanisms of chronic obstructive pulmonary disease [22], cancer, and other diseases [23–26]. In addition, network medicine-related algorithms, such as the connectivity map (CMap) and the search for off-label drugs and networks (SAveRUNNER), can be used to predict the link between diseases and drugs, significantly shortening the development cycle of new drugs [27].

In this study, we aimed to explore potential immune-related biomarkers in DN and elucidate the underlying mechanisms using bioinformatic approaches. By identifying the status of immune cell infiltration and immune-related biomarkers using bioinformatic approaches, new diagnostic and therapeutic targets can be identified for patients with DN.

## Material and methods

### Data acquisition and preparation

Five DN-related gene datasets, GSE96804 [28], GSE111154 [29], GSE104948-GPL22945 [30], GSE104948-GPL24120 [30], and GSE142025 [31] were obtained from the Gene Expression Omnibus (GEO). The details of the gene datasets are presented in Table 1. Four microarray datasets (GSE96804, GSE111154, GSE104948-GPL22945, and GSE104948-GPL24120) were merged, normalized, and utilized as the training cohort, and the RNA-sequencing gene dataset, GSE142025, was used as the test cohort. Probes with missing expression values were eliminated, and the average expression value was obtained when different probes pointed to the same gene. The batch effects were eliminated by employing the surrogate variable analysis (SVA) algorithm in the R environment [32]. Additionally, two-dimensional principal component analysis (PCA) was used to evaluate the distribution patterns in DN and normal samples and the microarray datasets.

**Table 1.** The information of GEO datasets in this study

GEO series	DN	Normal	Tissue	Platforms	Data type
GSE96804	41	20	glomeruli	GPL17586[HTA-2_0] Affymetrix Human Transcriptome Array 2.0	Train
GSE111154	4	4	glomeruli	GPL17586[HTA-2_0] Affymetrix Human Transcriptome Array 2.0	Train
GSE104948-GPL22945	7	18	glomeruli	GPL22945[HG-U133_Plus_2] Affymetrix Human Genome U133 Plus 2.0 Array	Train
GSE104948-GPL24120	5	3	glomeruli	GPL24120[HG-U133A] Affymetrix Human Genome U133A Array	Train
GSE142025	28	9	glomeruli	GPL20301Illumina HiSeq 4000 (Homo sapiens)	Test

DN, Diabetic Nephropathy.

### Differentially expressed immune-related genes (DEIRGs) screening

We obtained 3046 immune-related genes from Immport [33], TISIDB [34] and InnateDB [35], which are comprehensive databases that curate immune-related genes from research articles, books, and digital resources. We then intersected these immune-related genes in the training cohort. Ultimately, 1980 immune-related gene expression profiles were acquired. DEIRGs between the diabetic nephropathy and control groups were then analyzed by using the *‘limma’* package in R [36]. The cutoff criteria for DEIRG identification were |log_2_-fold change (FC)| ≥ 1 and Benjamini & Hochberg adjusted *p*-values < 0.05.

### Enrichment analysis of pathways and biological functions

The Database for Annotation, Visualization, and Integrated Discovery (DAVID) is a bioinformatics platform for the annotation and assessment of biological functions of genes [37]. Functional enrichment analysis was performed using DEIRGs, including the Kyoto Encyclopedia of Genes and Genomes (KEGG) and Gene Ontology (GO) databases using DAVID v6.8. GO analysis is a commonly used bioinformatics tool to identify biological processes in terms of molecular function (MF), biological processes (BP), and cellular components (CC), and to perform gene annotation [38]. The KEGG pathway database includes a variety of biochemical pathways and is a resource for understanding the advanced functions and utilities of biological systems [39]. The enrichment terms with Benjamini and Hochberg adjusted *p*-values < 0.05, were considered statistically significant.

### Evaluation of infiltrated immune cells

To explore the association between infiltrated immune cells and diabetic nephropathy, data on the proportions of the 22 immune cells in the standardized training dataset were obtained using the ‘cell-type identification by estimating relative subsets of RNA transcripts’ (CIBERSORT) algorithm. The immune cell infiltration matrix only included samples with *p* < 0.05. The proportions of infiltrated immune cells in each sample and each group were visualized in boxplots and violin plots, respectively. We selected immune cells that were significant (*p* < 0.05) between the two groups in the matrix for further analysis.

### Identification of significant modules with immune infiltration characteristics using WGCNA

To further understand the association between immune cells and their related gene expression profiles, we constructed a weighted co-expression network (WGCNA) and identified the significant gene modules related to the infiltrated immune cells. Before we utilized WGCNA, the gene expression matrix and the immune cell infiltration profile (which were acquired previously), were combined as one matrix for further analysis. We constructed the WGCNA network with the freely accessible R package, ‘*WGCNA’* [40].

In this study, we analyzed the combined matrix to construct gene co-expression networks that were associated with immune cell phenotypes. Obvious outliers were removed from the data, and a correlation matrix was constructed for all genes using Pearson’s correlation analysis. The co-expression network was constructed using a one-step method. We set a soft threshold R^2^ of 0.9, according to the criterion of scale-free topology [40], and an average linkage hierarchical clustering approach was used to classify genes into several co-expression modules.

Module membership (MM) and gene importance (GS) revealed the correlation between co-expressed genes and immune cell characteristics. Genes with higher MM and GS values indicated that these genes were more strongly correlated with modules and clinical characteristics, respectively. The genes with the highest immune cell correlation were extracted for further study (GS > 0.5; MM > 0.5). Candidate biomarkers were identified by cross-linking the genes obtained from the WGCNA and DEIRG analyses.

### PPI network construction and critical immune-related biomarker identification

To demonstrate the functional interactions among proteins, the overlapping genes from the WGCNA and DEIRG analyses were utilized to construct the PPI network, which was built using the STRING online platform (https:// string-db.org) [19] by setting the interaction score at high confidence (0.700). Furthermore, we identified significant gene clusters and hub immune-related genes using the Molecular Complex Detection (MCODE) algorithm in Cytoscape software [41]. We explored key immune-related biomarkers by applying the ‘*GOSemSim*’ package in R software to score the semantic similarity of GO terms in the gene clusters [42].

### Transcription factors (TFs)-microRNA (miRNA)-messengerRNA (mRNA) network construction

MicroRNAs and TFs control gene regulation. Therefore, further research on the regulatory relationship between TFs and miRNAs can elucidate the underlying mechanisms of immune-related gene markers in DN. The MIENTURNET (http://userver.bio.uniroma1.it/apps/mientumet/) web tool was used to assess miRNA-mRNA interactions [43]. We uploaded the hub immune-related genes to MIENTURNET and obtained miRNA-mRNA interactions. Furthermore, for the purpose of finding regulatory relationships between TFs and miRNAs, these miRNAs were input into the TransmiR platform [44], which is a database for TF-miRNA regulation. Finally, a TF-miRNA-mRNA network was constructed by merging the miRNA-mRNA and TF-miRNA interactions.

### Correlation analysis between immune-related biomarkers and infiltrated immune cells

We performed a Pearson correlation analysis on the key immune-related DN markers and infiltrating immune cells, and the results were visualized. The absolute value of correlation coefficient (r) greater than 0.5 was considered have positive or negative correlation.

### Validation of biomarkers in the testing cohort

To verify the biomarkers, we analyzed them based on the GSE142025 RNA-seq dataset. First, boxplots showed the differences in expression between the DN and normal samples. Then, we calculated the area under the curve (AUC) to assess the diagnostic value of these genes. If a gene had a high expression value in the DN sample (upregulated in the sample), then its AUC would be greater than 0.5; otherwise, it was < 0.5. A larger |AUC-0.5| value indicated that the gene could be distinguished between DN and control samples.

### Verification of the clinical relevance of biomarkers and the prediction of drug interactions

The clinical relevance of these genetic markers in patients with DN was explored using the Nephroseq database (https://www.nephroseq.org/) [45]. Nephroseq is an internet-based free access platform that includes a variety of human renal disease clinical and gene expression data sets that have been collected and managed by a team of experienced data scientists, bioinformaticians, and nephrologists, and allows researchers to conduct comprehensive data mining. We analyzed the correlation between hub genes and the glomerular filtration rate (GFR) in patients with DN based on the Woroniecka Diabetes Dataset in the Nephroseq database. Statistical significance was set at *p* < 0.05.

Given that the existing treatments for DN are not fully satisfactory, there is a need to propose novel tactics and develop new therapeutic approaches. The Connectivity Map (CMap; https://clue.io/) is a public database that collects expression profiles of cultured human cells treated with small molecules that have previously been used to explore drug mechanisms and identify new potential drugs [46]. DEIRGs were uploaded to the CMap online database to explore potential drugs for the treatment of DN. Enrichment scores ranged from −100 to 100, and the results were selected based on the magnitude of the correlation coefficient scores, with negatively correlated small molecule compounds being selected. After acquiring the results of CMap analysis, compounds with a mean coefficient of < −90 were selected and ranked according to their correlation scores. All cell lines provided by CMap were preserved in this study.

## Results

We screened DEIRGs and analyzed their biological enrichment to reveal the underlying immunological mechanisms in DN. Differentially expressed immune-related genes (DEIRGs) in multiple microarray glomerular datasets were identified. The proportion of infiltrated immune cells was calculated using the ‘cell-type identification by estimating relative subsets of RNA transcripts’ (CIBERSORT) algorithm. Key biomarkers and their functional enrichment were correlated with the pathogenesis and progression of DN. The biomarkers were verified using a test cohort and clinical databases, and therapeutic molecules related to the DEIRG were identified in DN.

### Data preprocessing

There were 45 control tissue samples and 57 DN glomeruli tissues in the GSE96804, GSE111154, GSE104948-GPL22945, and GSE104948-GPL24120 datasets. The clinical characteristics of the datasets are shown in Table S1.

**Figure 1.**
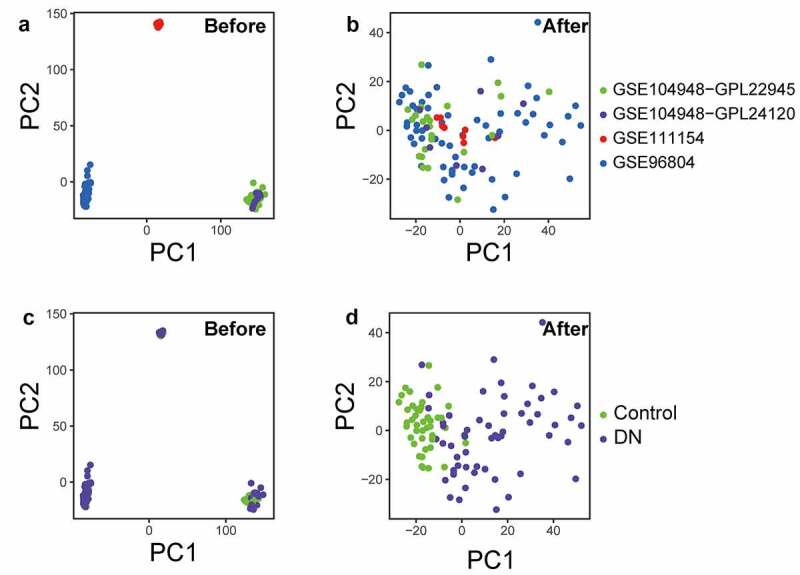
Samples PCA clustering plots for before and after calibration. (a, b) PCA Figures display inter-batch differences removed before and after correcting for GSE96804, GSE111154, GSE104948-GPL22945, and GSE104948-GPL24120, respectively. (c, d) Figures show batches differences of PCA cluster for DN and control samples before and after correction. PCA, principal components analysis. DN, diabetic nephropathy

The inter-batch difference was removed from the gene expression matrix after merging the datasets. The Q-Q plots and boxplots show that the inter-batch differences were removed (Figure S1). Before and after standardization of the training cohort, PCA results demonstrated that the batch effects in different datasets were eliminated (Figure 1(a,b)), and standardization resulted in a more pronounced clustering of samples from the DN and normal groups (Figure 1(c,d)), indicating that the sample sources were reliable.

### DEIRGs identification and biological enrichment

We screened DEIRGs and analyzed their biological enrichment to reveal the underlying immunological mechanisms in DN.

**Figure 2.**
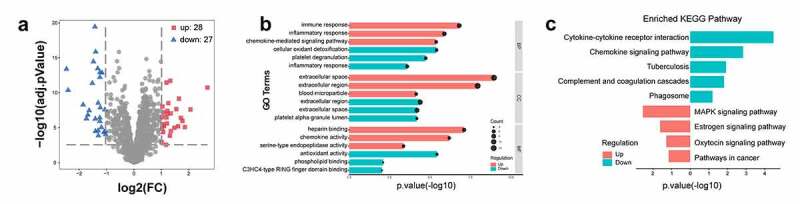
The DEIRGs and their functional enrichment results. (a) DEIRGs volcano plot; red represents up-regulated DEIRGs, and blue represents down-regulated DEIRGs. (b) GO biological function enrichment analysis for DEIRGs (c) KEGG pathway enrichment analysis in DEIRGs. DEIRGs, differently expressed immune-related genes. DN, diabetic nephropathy. DEIRGs: differently expressed immune-related genes. GO: gene ontology. KEGG: Kyoto Encyclopedia of Genes and Genomes

After normalization and annotation of the training cohort with 45 control tissue samples and 57 DN glomeruli tissues, DEIRGs were identified. With |(logFC)| ≥ 1 and adjusted *p*-values > 0.05, a total of 55 significant DEIRGs were found in the DN group compared to the normal samples, of which 28 were upregulated and 27 were downregulated. Volcano and heatmap plots of the DEIRGs are shown in Figure 2(a), S2. The list of DEIRGs is shown in Table S2.

GO analysis showed that most upregulated genes were particularly enriched in BP, including the immune response, inflammatory response, and chemokine-mediated signaling pathway (Figure 2(b), Table S3). Major enrichment in CC included extracellular space, extracellular region, and blood microparticles (Figure 2(b), Table S3). Primary enrichment in MF consisted of chemokine activity, heparin binding, and serine-type endopeptidase activity (Figure 2(b), Table S3). KEGG pathway analysis revealed that the upregulated DEIRGs were mainly enriched in cytokine-cytokine receptor interaction and chemokine signaling pathways (Figure 2(c), Table S4).

However, the results of GO terms for biological processes showed that the downregulated DEIRGs were mainly concentrated in cellular oxidant detoxification, platelet degranulation, and the inflammatory response (Figure 2(b), Table S5). The enriched GO terms for CC of downregulated DEIRGs included the extracellular region and extracellular space (Figure 2(b), Table S5). In addition, enriched GO terms for MF revealed that downregulated DEIRGs were mainly involved in antioxidant activity and phospholipid binding (Figure 2(b), Table S5). Moreover, downregulated DEIRGs were significantly enriched in pathways such as the MAPK, estrogen, and oxytocin signaling pathways (Figure 2(c), Table S4).

### Immune infiltration analysis

**Figure 3.**
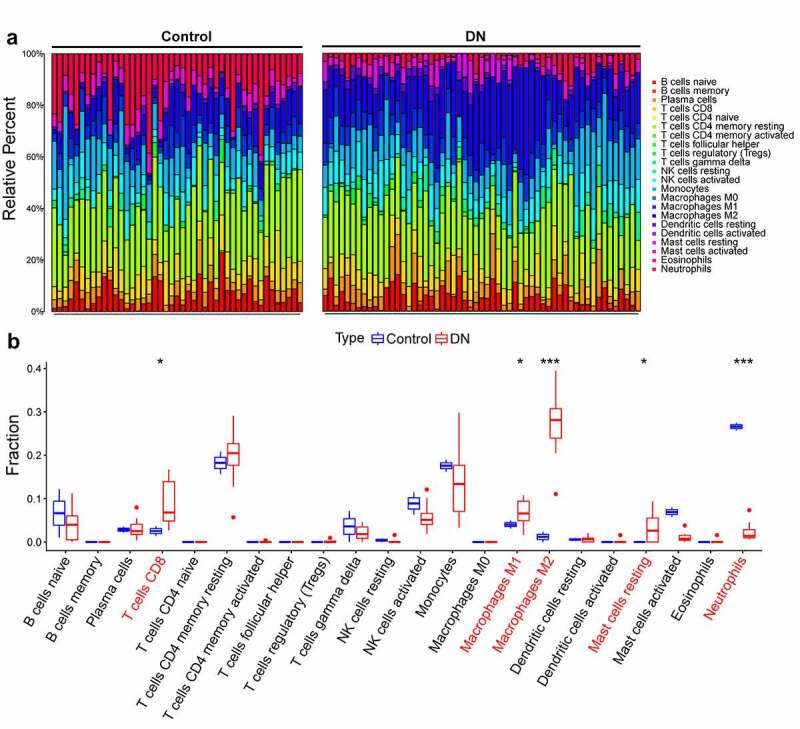
Assessment of immune infiltration. (a) Bar plot displays the proportion of infiltrated immune cells between DN and control groups. (b) box plot for difference immune cell infiltration between DN and normal samples (Wilcoxon’s test; *, p < 0.05; ***, p < 0.001). The horizontal coordinate represents the variety of infiltrated immune cells, and the vertical axis represents the fraction of infiltrated immune cell. DN, diabetic nephropathy

Due to technical limitations, the immune infiltration of DN was not fully elucidated. Using the CIBERSORT algorithm, we explored the differences in immune infiltration between DN and normal glomerular tissue. Compared with normal tissues, DN tissues generally contained a higher proportion of T cells CD8, Macrophages M1, Macrophages M2, and resting mast cells, whereas the proportion of neutrophils was lower (Figure 3(a), Figure 3(b)).

### Identification of immune-related gene modules using WGCNA

Using the WGCNA algorithm, genes associated with infiltrated immune cells were explored. A total of 11,221 genes were included in the WGCNA analysis, and the sample clustering results showed good consistency within groups and significant differences between groups (Figure S3A, S3B). The topology analysis showed that 10 was the minimal soft threshold power above the scale-free topology fit index of 0.9 (Figure S3C). After clustering, the genes were divided into 11 color-coded modules (Figure S3D), of which the genes in the gray module were those that could not be classified.

**Figure 4.**
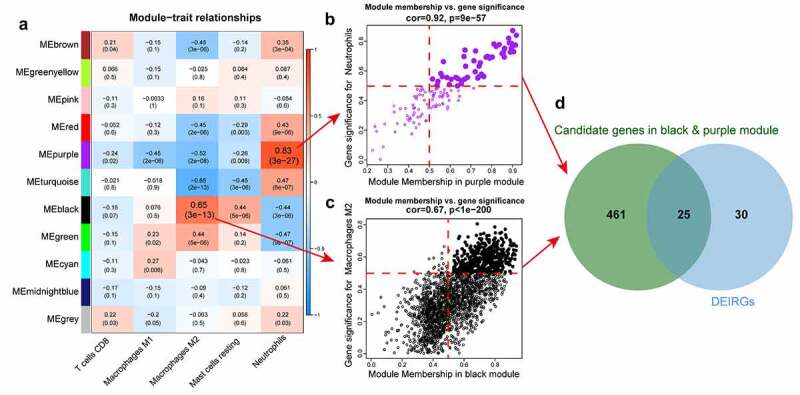
Screening candidate immune-related diabetic nephropathy biomarkers by using WGCNA. (a) Correlation between the gene module and infiltrated immune cell. The correlation coefficient in each cell represented the correlation between the gene module and the infiltrated immune cell, which decreased in color from red to blue. The corresponding *p*-value is also annotated. (b, c) Scatter plots depict the relationship between module membership (MM) and gene significance (GS) in black and purple module. Genes with MM>0.5 and GS>0.5 are labeled with solid dots. (d) Venn diagram shows the biomarkers intersected with DEIRGs and candidate genes in black and purple module. WGCNA, weighted correlation network analysis

We computed the module-trait correlation factors (Figure 4(a)). The black module (r = 0.65, p = 3E-13) had the highest correlation with the macrophage M2 trait, while the purple module had the best correlation with the neutrophil trait (r = 0.83, p = 3E-27). The GS and MM values for the two modules are presented in scatter plots, and genes with MM > 0.5, and GS > 0.5 were selected as candidate genes (Figure 4(b), 4(c); Table S6). Twenty-five overlapping genes from DEIRGs and candidate genes in the black and purple modules were retained for subsequent analysis (Figure 4(d)).

### PPI network construction and critical immune-related biomarker identification

**Figure 5.**
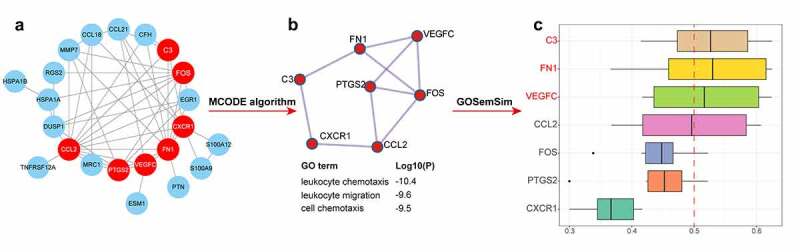
Construction of PPI network and identification of hub genes. (a) The PPI network constructed by STRING. Genes labeled red color are candidate hub genes chosen by MCODE algorithm. (b) PPI network and functional enrichment of candidate hub genes identified by MCODE algorithm. (c) The bar plot displays functional similarity analysis of candidate immune-related biomarkers, with the abscissa as the similarity score. PPI, protein-protein interaction. MCODE: molecular complex detection

To understand protein function, 25 candidate key biomarkers were obtained from the intersection of the DEIRGs and candidate genes in the black and purple modules. The gene list was uploaded to STRING, setting the interaction score at a high confidence level (0.700). Then, a PPI network with 22 nodes and 50 edges was constructed, where each node represented a protein, and each edge represented an interaction between proteins (Figure 5(a)). By applying the MCODE algorithm, a densely connected gene cluster was identified, which included seven key genes whose GO enrichment analysis was mainly enriched in leukocyte chemotaxis, leukocyte migration, and cell chemotaxis (Figure 5(b)).

To further mine the key genes, we used the ‘*GOSemSim*’ package in R to calculate the GO semantic similarity of these seven genes. The higher the semantic similarity, more important the role that the gene plays in the function. Our results revealed that FN1, C3, and VEGFC had higher functional similarities (similarity score > 0.5) and immune-related DN hub genes (Figure 5(c)).

### Construction of the TF-miRNA-mRNA regulatory network

**Figure 6.**
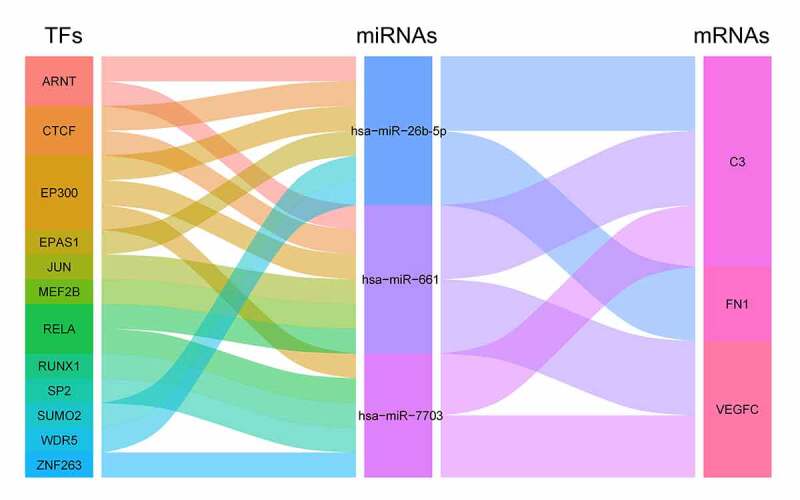
The alluvial diagram demonstrated the ‘TF-miRNA-mRNA’ regulatory network constructed by 12 TFs, 3 miRNAs, and 3 mRNA targets. TF, transcription factor

We uploaded the immune-related gene markers FN1, C3, and VEGFC into the MIENTURNET platform to search for interacting microRNAs (miRNAs). Then, we filtered the microRNAs by setting the species to ‘*Homo sapiens*’ and the tissue to ‘kidney’, and obtained seven regulating microRNAs (Table S7). These miRNAs were input into the TransmiR platform, which is a database for TF-microRNA regulation. After merging miRNA-mRNA and miRNA-TF regulation, we constructed a TF-miRNA-mRNA network, which included three miRNAs, three mRNAs, and twelve transcription factors (Figure 6).

### Association analysis of diagnostic biomarkers with infiltrating immune cells

**Figure 7.**
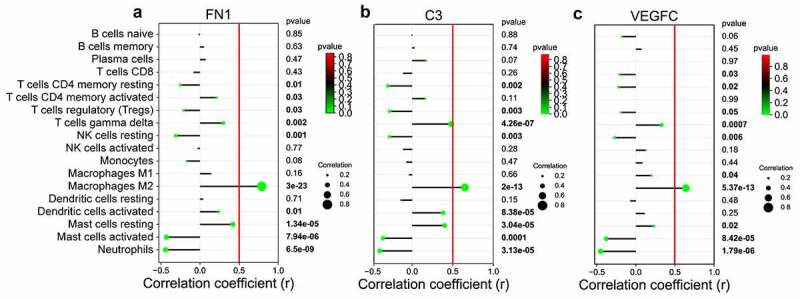
Lollipop figures for the correlation between candidate immune-related biomarkers of DN and infiltrated immune cells. (a) FN1 is positively correlated with macrophages M2 cells. (r = 0.79, p = 3.00E-23). (b) Correlation analysis presents that C3 has positive relationship with macrophages M2 cells. (r = 0.65, p = 2.0E-13). (c) Positive association was also found in the relationship between VEGFC and macrophages M2 cells (r = 0.64, p = 5.37E-13). DN: diabetic nephropathy

We wanted to determine whether these hub genes were related to immune cell infiltration using Pearson’s correlation analysis. Correlation analysis showed that FN1 had a positive relationship with macrophages M2 (r = 0.73, p = 1.30E-18); C3 had a positive correlation with macrophage M2 cells (r = 0.63, p = 1.74E-12), and VEGFC and macrophages M2 cells were also positively correlated (r = 0.62, p = 2.25E-12) (Figure 7, Table S8).

### Validation of biomarkers based on the test cohort

**Figure 8.**
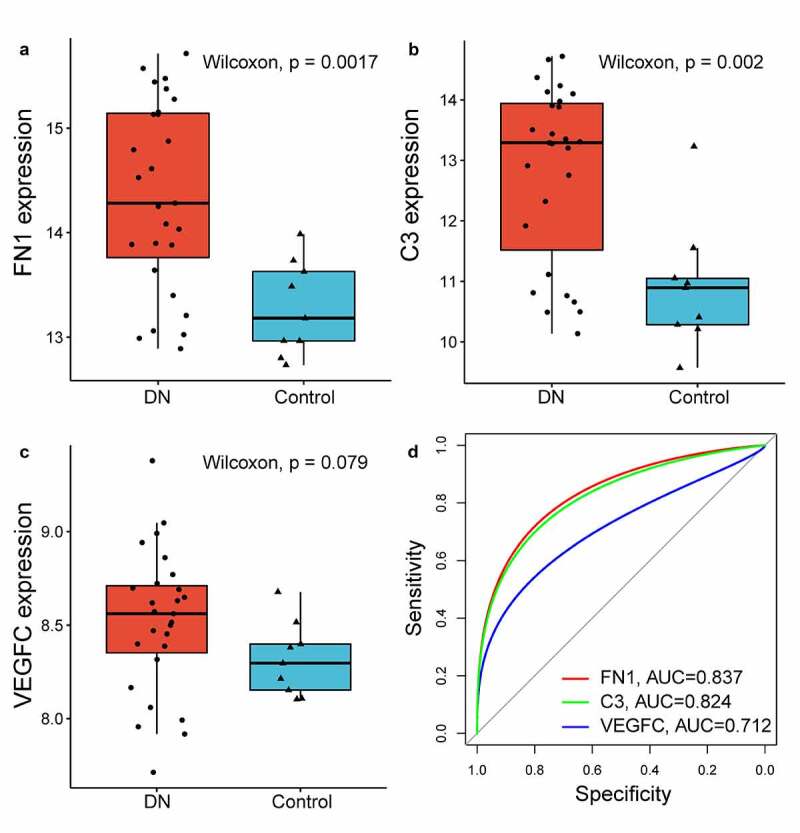
Validation of immune-related hub genes based on RNA-seq dataset GSE14025. (a-c) Gene expression levels of hub genes between DN and control group. (d) The receiver operating characteristic curve of hub genes in GSE14025. DN: diabetic nephropathy

To test the applicability and robustness of these biomarkers, we validated them in the test cohort. The expression levels of these biomarkers in the test cohort were confirmed. Two markers (FN1 and C3) were statistically higher in the DN group than in the normal group (Figure 8(a,b)). However, VEGFC was not significantly different between the DN and normal groups (Figure 8(c)). The receiver operator characteristic (ROC) curve analysis illustrated that FN1 and C3 had large AUC values (0.837 and 0.824, respectively), which indicated that FN1 and C3 had the strongest predictive ability among the four biomarkers (Figure 8(d)).

### Verification of the clinical relevance of biomarkers and prediction of drugs

**Figure 9.**
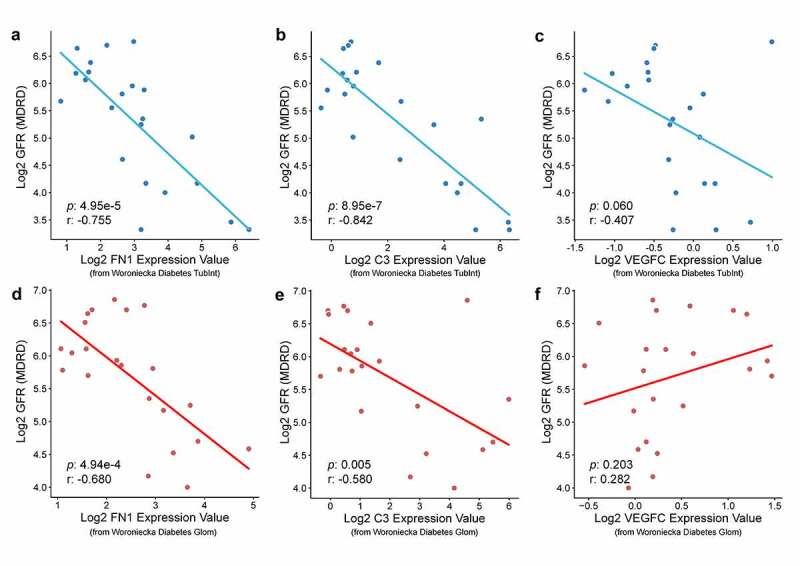
Pearson correlation analysis of GFR and target genes. FN1 is negatively related to GFR in diabetic nephropathy tubule samples(a) (r = −0.755, p = 4.95E-5) and glomerulus samples (d) (r = −0.68, p = 4.94E-4). C3 has also negatively relationship with GFR in nephropathy tubule samples(b) (r = −0.842, p = 8.95E-7) and glomerulus samples (e) (r = −0.58, p = 0.005). The expression of VEGFC has not statistically significance with GFR in patient’s tubule samples(c) (r = −0.407, p = 0.06) and glomerulus samples (f) (r = 0.282, p = 0.203). GFR, glomerular filtration rate. DN: diabetic nephropathy

With regard to the correlation between biomarkers and clinical features, FN1 and C3 were found to be significantly negatively correlated with GFR in DN glomerulus (r = −0.68, p = 4.94E-4 and r:-0.58, p = 0.005) and tubule (r = −0.755,p = 4.95E-5 and r = −0.842, p = 8.95E-7) samples based on Woroniecka diabetes data in the Nephroseq platform, suggesting a pathogenic role of biomarkers (Figure 9(a), 9(d); Figure 9(b, 9(e)). However, VEGFC showed a statistically significant correlation with GFR in DN (Figure 9(c), 9(f)).

**Figure 10.**
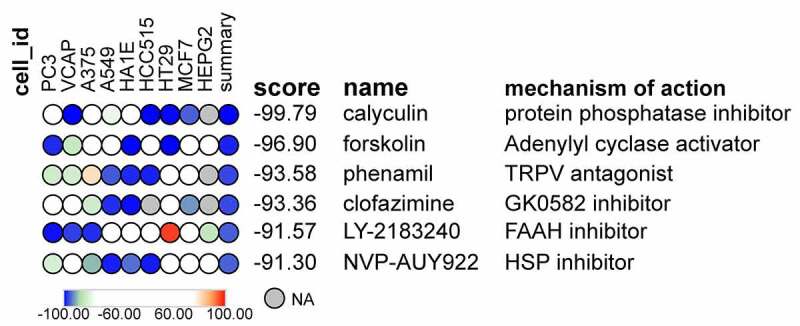
Potential therapeutic molecular compounds predicted by connectivity map. Compounds with a mean coefficient less than −90 were selected and ranked according to the correlation score. Note: PC3, human prostatic carcinoma cell line. VCAP, vertebral cancer of the prostate cell line. A375, human melanoma cell line. A549, adenocarcinomic human alveolar basal epithelial cells. HA1E, human embryonic kidney cell line. HCC515, human lung cancer cell line. HT29, human colorectal adenocarcinoma cell line. MCF7, breast cancer cell line. HEPG2, human hepatocyte carcinoma cell line

DEIRGs were compared to the reference gene list in the connectivity map database. Twenty-eight upregulated DEIRGs and twenty-seven downregulated DEIRGs were imported into the connectivity map to map potential agents. Compounds with a mean coefficient of < −90 were selected and ranked according to their correlation scores. The results showed that there were six chemical compounds, including calyculin, forskolin, phenamil, clofazimine, LY-2,183,240, and NVP-AUY922 that were negative and < −90. These findings indicated that the overall perturbation of DN by these chemical compounds was opposite to that of the differentially immune-related genes. Thus, these compounds or their analogs may potentially play antagonistic roles in DN (Figure 10).

## Discussion

In this study, we explored infiltrated immune cells and related biomarkers in DN using bioinformatic methods. The conventional view of the pathogenesis of DN maintains that the main agonistic factors are hemodynamic and metabolic disorders caused by the hyperglycemic environment [47]. However, mounting evidence suggests that immune cell infiltration and inflammation play an essential role in the etiopathogenesis of DN [48]. According to the analysis of functional enrichment, we found that most upregulated DEIRGs were enriched in the extracellular matrix and participated in the biological processes of immune and inflammatory responses. Using single nuclear RNA sequencing technology, a recent study found that immune cell infiltration and aberrant angiogenesis are early signs of DN [49]. Hyperglycemia can activate macrophages and cytokines, which leads to the accumulation and infiltration of immune cells in the kidney tissues of patients with DN [50,51]. Therefore, it is not surprising that cytokine-cytokine receptor interactions were among the most active pathways in our KEGG analysis.

Moreover, using the CIBERSORT algorithm, we found that macrophages were the most infiltrative immune cells in DN. Previous studies have reported that the accumulation of macrophages can be discovered in the renal tissue of patients with DN and portend renal function decline [52,53]. Macrophage infiltration is an important feature of DN [54], and the high glucose and glycosylation end products in the DN environment promote the recruitment and migration of macrophages, which release inflammation-promoting factors, leading to kidney injury and fibrosis [55]. The increase in macrophages is also associated with upregulated ICAM-1 and MCP-1 by kidney tubular cells in response to hyperglycemia and advanced glycation end products (AGEs) [56,57]. Macrophages are divided into M1 and M2 macrophages. M1 macrophages secrete excessive amounts of pro-inflammatory and chemotactic factors that promote an inflammatory response and damage normal kidney tissue [58]. However, the role of M2 macrophages in renal tissue fibrosis remains controversial, as they can differentiate into fibroblasts and contribute to the proliferation and activation of myofibroblasts, as well as participate in the repair and reconstruction of DN kidney injury by phagocytosing damaged cells, downregulating the expression of inflammatory cytokines and chemokines, and inhibiting the toxic effects of T cells [59]. It has been found that macrophages have an M1 phenotype in the early stages of kidney injury and an M2 phenotype in the repair stage, and M1 macrophages can be converted to an M2 phenotype over time [60]. Promoting M2 macrophages and reducing the M1 phenotype could be promising therapeutic strategies for DN. Current research shows that neutrophils are mainly involved in acute kidney injury [61], but their role in chronic DN remains unclear. In our study, we found that the proportion of neutrophils was relatively higher in normal samples, which may be a potential limitation of the CIBERSORT algorithm, because the higher proportion of macrophages in patients with DN makes the proportion of other immune cells, including neutrophils, appear lower. Mast cells are reported to increase in patients with DN, and their levels are related to serum creatinine levels [62]. Although there is evidence of stenosis if T cells are engaged in the development of DN, limited animal experiments have found that CD6+ and CD4 + T cells are moderately increased in type 2 diabetes patients and are correlated with proteinuria [63]. Other immune cells did not demonstrate substantial differences in our study, and their roles in DN require further exploration.

Next, we combined multiple bioinformatic approaches, including WGCNA and computational biology algorithms such as MCODE and GOSemSim, to identify gene markers and found FN1, C3, and VEGFC to be candidate markers. TFs and miRNAs regulate mRNA gene expression. Additionally, miRNAs and TF could alter the expression of each other. We constructed the TF-miRNA-mRNA network by using bioinformatics tools, which revealed that miRNAs (has-miR-26b-5p, has-miR-661, has-miR-7703) and TF (ARNT, CTCF, JUN and so on) might regulate the gene expression in the DN. Aryl hydrocarbon receptor nuclear translocator (ARNT) is a transcription factor that has been reported to play a vital role in regulating glycolysis, angiogenesis, and apoptosis. Low-dose tacrolimus exerts antifibrotic, renoprotective effects in a model of renal fibrosis via ARNT-mediated transcription of bone morphogenetic protein receptor type 1A [64]. It is reported that CTCF can regulate miR-185-5p/NPHS2 axis with a net effect of alleviating renal interstitial fibrosis in chronic kidney disease [65]. In our study, only FN1 and C3 showed statistical significance in the test cohort and clinical database, so they were ultimately identified as immune-related DN biomarkers. In our study, fibronectin 1 (FN1) was found to be highly expressed in patients with DN and was positively correlated with macrophages M2. It is known that FN1 is an accumulation constituent of the extracellular matrix in the case of hyperglycemia and plays an essential role in renal fibrosis [66,67]. FN1 encodes fibronectin, a glycoprotein present in plasma and in extracellular matrix, which is heavily upregulated in inflamed tissues and in vitro can serve as a substrate for leukocyte migration [68], and may prove beneficial in promoting T cell accumulation in tissues and enhancing local immunity to infection or cancer [69]. Further verification showed that FN1 expression is related to the decline in GFR in patients with DN. C3, which plays a central role in the activation of the complement system, was overexpressed in patients with DN and was negatively correlated with GFR in this study. It has been shown that complement synthesis is closely associated with the development and progression of renal disease and that C3 secreted by macrophages leads to IL-17A-mediated inflammatory cell infiltration in renal tissue. C3 further promotes M1 polarization of macrophages, promotes the expression of inflammatory factors and exacerbates renal interstitial fibrosis [70,71].Our KEGG results also showed that the complement and coagulation cascade pathways were involved in the pathogenesis of DN, which is consistent with existing knowledge [5]. Researchers have suggested that complement C3 is activated in podocytes and renal tubules in animal models of diabetic nephropathy, causing fibrosis and renal dysfunction, and that administration of C3 receptor blockers protects diabetic nephropathy podocytes from injury [72,73]. Large clinical studies have shown that C3 is involved in diabetic microangiopathy and is associated with the progression of diabetic nephropathy [74,75]. Moreover, patients with glomerular complement C3 deposition have worse clinical outcomes [76]. However, the best methods for targeting the immune system to prevent DN progression still need to be investigated. We identified six potential small-molecule compounds in our study using the connectivity map database. Of these compounds, forskolin has been proven to protect podocytes by inhibiting protein biosynthesis in a cAMP-dependent pathway [77]. Another study also revealed that forskolin may inhibit blood glucose levels and macrophage activation, thereby exerting antioxidant and anti-inflammatory effects in a diabetic rat model [78]. However, future studies would benefit from experimental validation to fully elucidate the mechanisms underlying immune-related biomarkers in DN.

## Conclusion

In summary, we identified the status of immune cell infiltration and immune-related biomarkers using bioinformatic approaches. FN1 and C3 were screened and found to be closely related to the pathogenesis and progression of DN, as well as macrophage infiltration.

Clinical database verification showed that they were positively correlated with the GFR. Six small-molecule compounds were identified as potential therapeutic agents. Further exploration of these immune cells and biomarkers may provide new diagnostic and therapeutic targets for patients with DN.

## Supplementary Material

Supplemental Material

## Data Availability

The authors confirm that the data supporting the findings of this study are available within the article.

## Highlights

1. FN1 and C3 were identified as the immune infiltration-associated biomarkers of DN
2. The infiltrated immune cell landscape of DN was demonstrated
3. Six potential therapeutic molecular compounds of DN were predicted
