# Supplementary material for: Identification of fibronectin 1 (FN1) and complement component 3 (C3) as immune infiltration-related biomarkers for diabetic nephropathy using integrated bioinformatic analysis
Source: Bioengineered. 2021 Aug 23;12(1):5386–401. doi: 10.1080/21655979.2021.1960766 (PMC8806822; doi:10.1080/21655979.2021.1960766)
Supplement: Supplemental Material [file KBIE_A_1960766_SM8755.zip › supplementary/Supplementary figures_revised.pptx]

## Slide 1
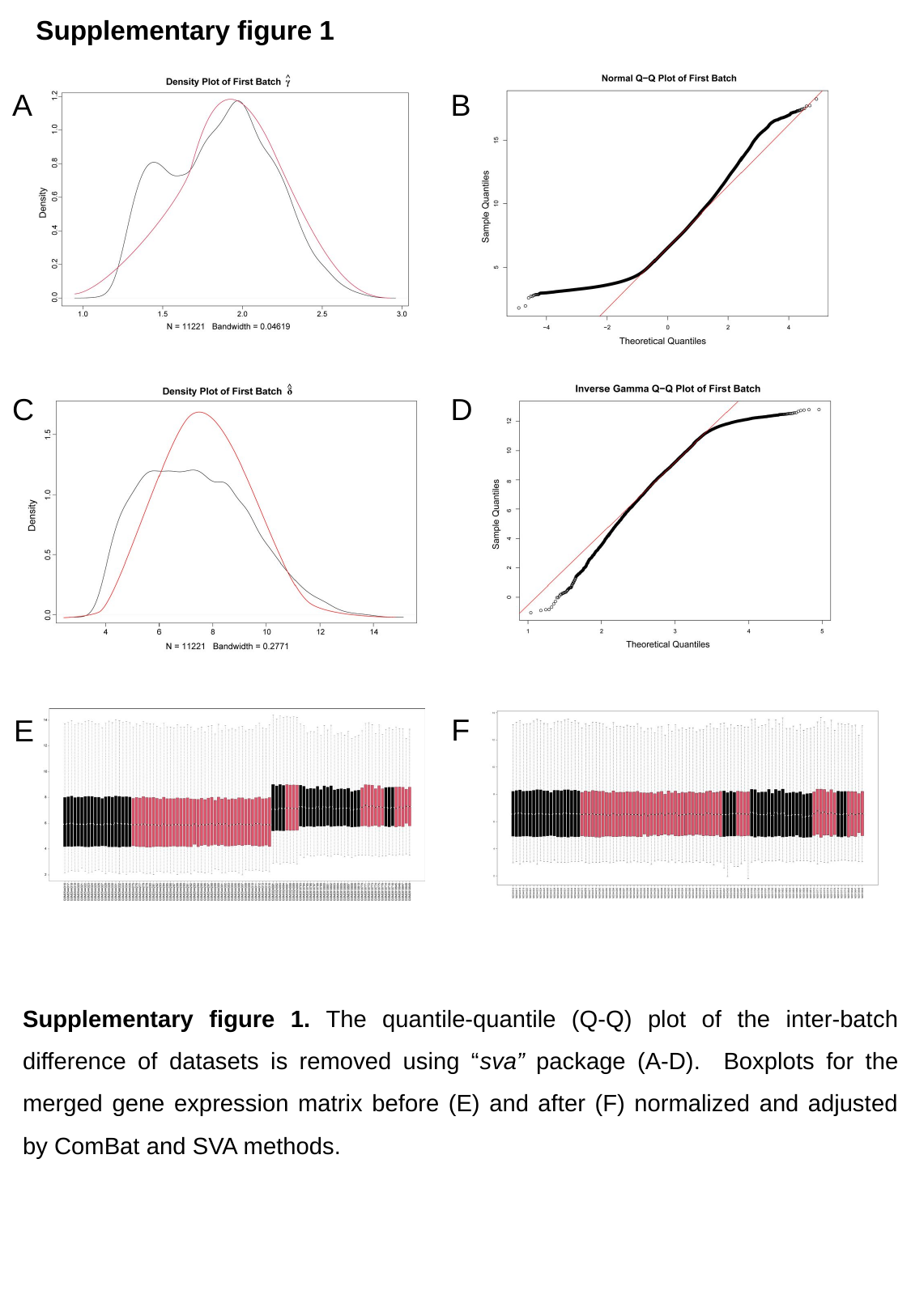

Supplementary figure 1
A
B
C
D
F
E
Supplementary figure 1. The quantile-quantile (Q-Q) plot of the inter-batch difference of datasets is removed using “sva” package (A-D). Boxplots for the merged gene expression matrix before (E) and after (F) normalized and adjusted by ComBat and SVA methods.

## Slide 2
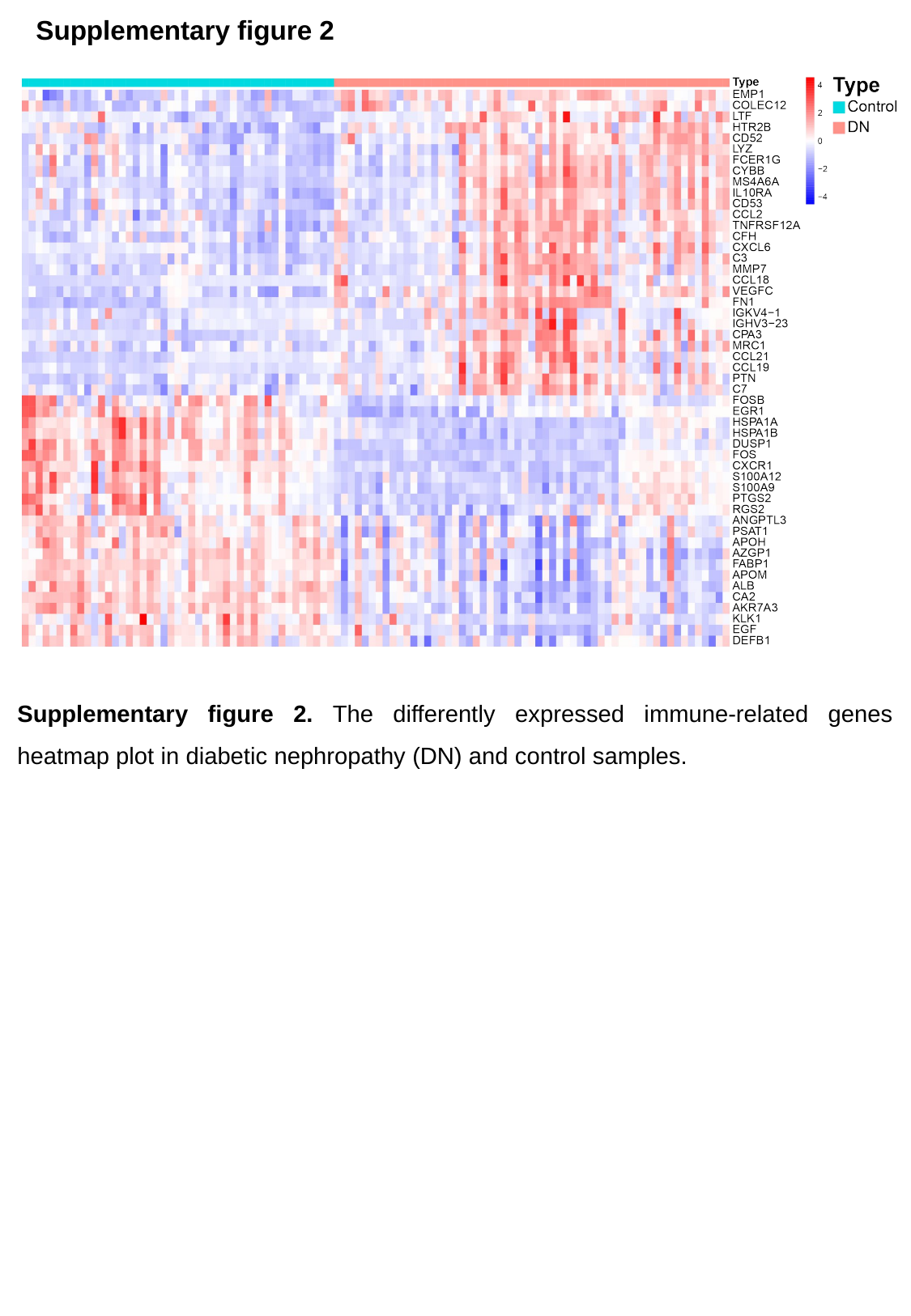

Supplementary figure 2
Supplementary figure 2. The differently expressed immune-related genes heatmap plot in diabetic nephropathy (DN) and control samples.

## Slide 3
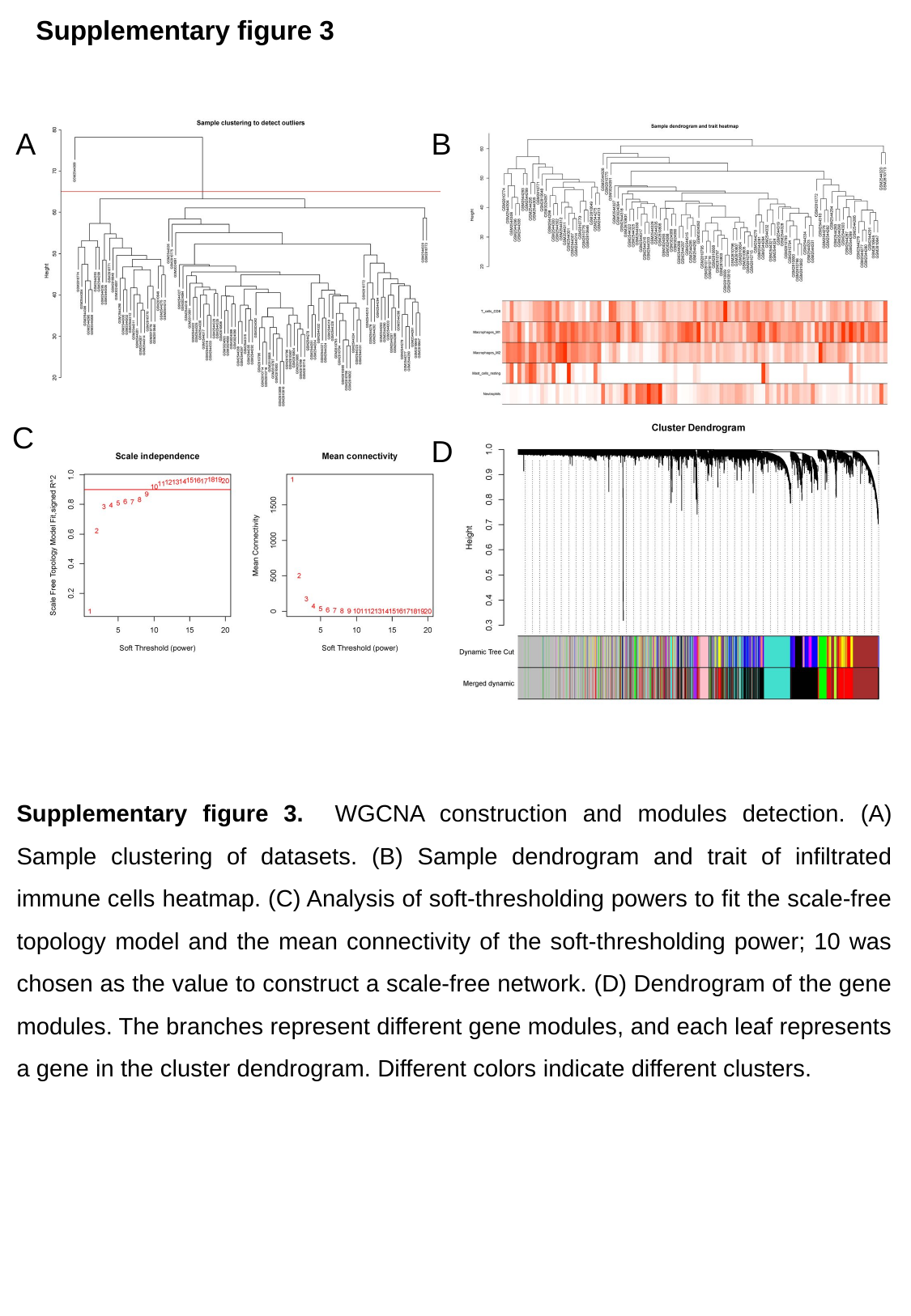

Supplementary figure 3
A
B
C
D
Supplementary figure 3. WGCNA construction and modules detection. (A) Sample clustering of datasets. (B) Sample dendrogram and trait of infiltrated immune cells heatmap. (C) Analysis of soft-thresholding powers to fit the scale-free topology model and the mean connectivity of the soft-thresholding power; 10 was chosen as the value to construct a scale-free network. (D) Dendrogram of the gene modules. The branches represent different gene modules, and each leaf represents a gene in the cluster dendrogram. Different colors indicate different clusters.
