# Supplementary material for: Identification of fibronectin 1 (FN1) and complement component 3 (C3) as immune infiltration-related biomarkers for diabetic nephropathy using integrated bioinformatic analysis
Source: Bioengineered. 2021 Aug 23;12(1):5386–401. doi: 10.1080/21655979.2021.1960766 (PMC8806822; doi:10.1080/21655979.2021.1960766)
Supplement: Supplemental Material [file KBIE_A_1960766_SM8755.zip › supplementary/Supplementary tables_revised.docx]

# Supplementary table 1. Available clinical characteristics of DN patients and the healthy control in this study.

|  | **GSE96804** | | **GSE142025** | | | **GSE111154** | | |
| --- | --- | --- | --- | --- | --- | --- | --- | --- |
| **Baseline** | **Control** | **DN** | **Control** | **DN** | **Baseline** | | **Control** | **DN** |
| **characteristics** | **(n=20)** | **（n=41）** | **(n=9)** | **(n=28)** | **characteristics** | | **(n=4)** | **(n=4)** |
| Male/female (% male) | 14/6(70) | 29/12(70.7) | 7/2 (77.7) | 18/10 (64.2) | Male sex, *n* | | 2 | 2 |
| Age (year) | 43.2±6.52 | 46.9±7.70 | 60.89±2.85 | 53.18 ±2.47 | Age in years, mean (range) | | 52 (24–65) | 64 (55–78) |
| Body mass index (kg/m2) | 21.6±2.52 | 24.8±1.82 | 23.38±1.39 | 25.57 ± 0.70 | Hypertension, *n* (%) | | 3 (75) | 4 (100) |
| Glycated hemoglobin A1c (%) | 5.21±0.58 | 6.78±1.82 | 5.68±0.29 | 8.455 ± 0.66 | BMI in kg/m^2^, mean (range) | | 27 (26–29) | 35 (22–43) |
| Systolic blood pressure (mm/Hg) | 123.2±18.2 | 134.2±15.8 | 128.0±1.80 | 141.6 ±4.38 | Terminal serum creatinine, mean, mg/dL | | 0.9 | 1.4 |
| Diastolic blood pressure (mm/Hg) | 75.3±10.2 | 84.8±8.2 | 75.67±2.26 | 84.41 ± 2.49 | Proteinuria (≥1 + by UA), % | | 50 | 50 |
| Total urinary protein (g/24hr) | － | 2.53±1.20 | 0.09±0.01 | 6.11 ± 0.97 | Hemoglobin A1c, mean, % | | 5.4 | 9.5 |
| eGFR (ml/min) | 100.23±10.5 | 63.16±22.12 | 117.7 ±8.62 | 63.79 ± 5.76 | eGFR, mean mL/minute | | >60 | >60 |

# Supplementary table 2. The DEIRGs between DN samples and normal samples.

| **Gene symbol** | **logFC** | **Ave Expr** | **t** | **P. Value** | **adj. P. Val** | **B** |
| --- | --- | --- | --- | --- | --- | --- |
| DUSP1 | -1.42224 | 8.004227 | -13.0276 | 1.87E-23 | 3.70E-20 | 42.7382 |
| CA2 | -1.3968 | 9.475013 | -11.2171 | 1.61E-19 | 1.42E-16 | 33.88259 |
| CYP27B1 | -1.47692 | 5.891722 | -10.3774 | 1.15E-17 | 3.54E-15 | 29.70171 |
| ESM1 | -1.31985 | 6.621815 | -9.86662 | 1.56E-16 | 3.44E-14 | 27.14903 |
| FOS | -2.46228 | 7.09981 | -9.81039 | 2.08E-16 | 4.12E-14 | 26.86791 |
| HSPA1A | -1.23578 | 9.990098 | -9.54761 | 7.96E-16 | 1.21E-13 | 25.55462 |
| AKR7A3 | -1.16443 | 7.038047 | -9.50271 | 1.00E-15 | 1.42E-13 | 25.33033 |
| HSPA1B | -1.2425 | 9.870873 | -9.18114 | 5.16E-15 | 6.01E-13 | 23.72566 |
| EMP1 | 1.331422 | 9.138825 | 8.907729 | 2.07E-14 | 1.95E-12 | 22.36472 |
| VEGFC | 1.193889 | 7.59771 | 8.749093 | 4.63E-14 | 3.53E-12 | 21.57711 |
| CXCR1 | -1.5178 | 6.072513 | -8.69516 | 6.08E-14 | 4.46E-12 | 21.30972 |
| FN1 | 2.686836 | 8.953497 | 8.396613 | 2.75E-13 | 1.81E-11 | 19.83405 |
| ALB | -2.40057 | 7.74868 | -8.18049 | 8.15E-13 | 4.36E-11 | 18.77119 |
| MS4A6A | 1.649418 | 6.928143 | 7.541555 | 1.96E-11 | 7.17E-10 | 15.66433 |
| EGF | -1.30308 | 6.628825 | -7.20528 | 1.02E-10 | 3.05E-09 | 14.05629 |
| HTR2B | 1.259837 | 6.993023 | 7.194971 | 1.07E-10 | 3.16E-09 | 14.00735 |
| FABP1 | -1.85389 | 10.63079 | -7.04238 | 2.24E-10 | 5.47E-09 | 13.28553 |
| IL10RA | 1.0397 | 6.65238 | 6.91578 | 4.12E-10 | 9.27E-09 | 12.69066 |
| APOH | -1.2265 | 6.03459 | -6.81564 | 6.66E-10 | 1.39E-08 | 12.2228 |
| CCL2 | 1.551783 | 11.22591 | 6.69271 | 1.20E-09 | 2.31E-08 | 11.65192 |
| C3 | 2.069838 | 7.561414 | 6.66488 | 1.37E-09 | 2.55E-08 | 11.52322 |
| AZGP1 | -1.05756 | 7.17313 | -6.64252 | 1.52E-09 | 2.76E-08 | 11.41996 |
| S100A12 | -1.6333 | 5.893638 | -6.57834 | 2.06E-09 | 3.61E-08 | 11.12435 |
| PTN | 1.221115 | 7.280336 | 6.541824 | 2.44E-09 | 4.03E-08 | 10.95667 |
| TNFRSF12A | 1.07263 | 9.418026 | 6.400013 | 4.76E-09 | 6.73E-08 | 10.30908 |
| CPA3 | 1.34556 | 4.983784 | 6.316625 | 7.02E-09 | 9.14E-08 | 9.931056 |
| CFH | 1.223491 | 10.71274 | 6.290731 | 7.91E-09 | 9.86E-08 | 9.814103 |
| S100A9 | -1.68047 | 9.04192 | -6.18069 | 1.32E-08 | 1.52E-07 | 9.319438 |
| CD52 | 1.032679 | 7.045685 | 5.997847 | 3.04E-08 | 3.17E-07 | 8.506305 |
| CD53 | 1.194664 | 8.400344 | 5.978125 | 3.33E-08 | 3.43E-07 | 8.419278 |
| FOSB | -1.24442 | 6.480152 | -5.95071 | 3.77E-08 | 3.77E-07 | 8.298518 |
| COLEC12 | 1.718615 | 6.756683 | 5.921332 | 4.31E-08 | 4.14E-07 | 8.169433 |
| EGR1 | -1.73017 | 9.494268 | -5.85613 | 5.78E-08 | 5.26E-07 | 7.884 |
| APOM | -1.40637 | 9.449343 | -5.83395 | 6.39E-08 | 5.67E-07 | 7.787269 |
| CCL21 | 1.7777 | 6.94272 | 5.661705 | 1.38E-07 | 1.09E-06 | 7.042312 |
| ANGPTL3 | -1.24096 | 6.948779 | -5.63901 | 1.53E-07 | 1.20E-06 | 6.945013 |
| CXCL6 | 1.208885 | 5.25315 | 5.444284 | 3.59E-07 | 2.52E-06 | 6.118623 |
| FCER1G | 1.041913 | 9.283678 | 5.377073 | 4.80E-07 | 3.20E-06 | 5.83704 |
| CYBB | 1.103038 | 8.055265 | 5.209935 | 9.84E-07 | 5.90E-06 | 5.145311 |
| C7 | 1.61493 | 10.63669 | 5.178115 | 1.13E-06 | 6.60E-06 | 5.015033 |
| MMP7 | 1.862356 | 10.33874 | 5.114497 | 1.47E-06 | 8.44E-06 | 4.755956 |
| PTGS2 | -1.18485 | 5.338564 | -5.08311 | 1.68E-06 | 9.41E-06 | 4.628828 |
| CCL19 | 1.480807 | 6.110718 | 5.057605 | 1.87E-06 | 1.03E-05 | 4.525869 |
| LTF | 1.029167 | 6.483877 | 4.983344 | 2.55E-06 | 1.37E-05 | 4.227843 |
| DEFB1 | -1.30583 | 14.45078 | -4.80709 | 5.27E-06 | 2.64E-05 | 3.531306 |
| RGS2 | -1.04537 | 10.29763 | -4.75571 | 6.50E-06 | 3.16E-05 | 3.331201 |
| CCL18 | 1.351208 | 4.636712 | 4.750113 | 6.65E-06 | 3.22E-05 | 3.309485 |
| IGF1 | -1.40108 | 8.788429 | -4.74097 | 6.90E-06 | 3.33E-05 | 3.274045 |
| MRC1 | 1.070697 | 7.243307 | 4.671164 | 9.14E-06 | 4.27E-05 | 3.004903 |
| PSAT1 | -1.08977 | 9.003695 | -4.63776 | 1.04E-05 | 4.79E-05 | 2.877031 |
| KLK1 | -1.03237 | 6.278912 | -4.45082 | 2.18E-05 | 9.07E-05 | 2.172507 |
| KNG1 | -1.29665 | 9.671647 | -4.33053 | 3.48E-05 | 0.000138 | 1.729467 |
| LYZ | 1.315683 | 10.55848 | 4.323508 | 3.57E-05 | 0.000141 | 1.703855 |
| IGKV4-1 | 1.016014 | 5.271536 | 3.636985 | 0.000434 | 0.001251 | -0.6539 |
| IGHV3-23 | 1.176222 | 6.206783 | 3.363542 | 0.001084 | 0.002798 | -1.50655 |

Ave Expr, average expression.

Adj. P. Val, adjusted P. value.

# Supplementary table 3. Top 5 upregulated DEIRGs GO annotation (BP term, CC term, MF term).

| **ONTOLOGY** | **ID** | **Description** | **Count** | **P. Value** | **List Total** | **Fold Enrichment** | **FDR** |
| --- | --- | --- | --- | --- | --- | --- | --- |
| BP | GO:0006955 | immune response | 9 | 1.54E-07 | 27 | 13.29 | 5.24E-05 |
| BP | GO:0006954 | inflammatory response | 8 | 1.28E-06 | 27 | 13.12 | 1.92E-04 |
| BP | GO:0071346 | cellular response to interferon-gamma | 5 | 1.69E-06 | 27 | 54.55 | 1.92E-04 |
| BP | GO:0070098 | chemokine-mediated signaling pathway | 5 | 4.09E-06 | 27 | 43.79 | 3.49E-04 |
| BP | GO:0006956 | complement activation | 5 | 9.21E-06 | 27 | 35.74 | 5.98E-04 |
| CC | GO:0005615 | extracellular space | 15 | 1.09E-09 | 28 | 7.24 | 5.79E-08 |
| CC | GO:0005576 | extracellular region | 15 | 1.11E-08 | 28 | 6.06 | 2.94E-07 |
| CC | GO:0072562 | blood microparticle | 5 | 7.03E-05 | 28 | 21.40 | 1.20E-04 |
| CC | GO:0009986 | cell surface | 7 | 1.17E-04 | 28 | 8.40 | 1.50E-04 |
| CC | GO:0070062 | extracellular exosome | 10 | 0.016382725 | 28 | 2.31 | 0.17 |
| MF | GO:0008201 | heparin binding | 7 | 7.71E-08 | 25 | 29.54 | 5.01E-06 |
| MF | GO:0008009 | chemokine activity | 5 | 6.38E-07 | 25 | 68.90 | 2.07E-05 |
| MF | GO:0004252 | serine-type endopeptidase activity | 5 | 4.26E-04 | 25 | 13.24 | 7.00E-03 |
| MF | GO:0048020 | CCR chemokine receptor binding | 3 | 4.81E-04 | 25 | 88.071 | 7.00E-03 |
| MF | GO:0031732 | CCR7 chemokine receptor binding | 2 | 0.002841496 | 25 | 675.24 | 0.03 |

BP，Biological process

CC，Cellular component

MF，Molecular function

# Supplementary table 4. KEGG pathway enrichment of upregulated and downregulated DEIRGs

| **ID** | **Term** | **Count** | **P. Value** | **List Total** | **Fold Enrichment** | **FDR** | **Type** |
| --- | --- | --- | --- | --- | --- | --- | --- |
| hsa04060 | Cytokine-cytokine receptor interaction | 7 | 3.37E-05 | 20 | 9.90 | 1.00E-03 | Upregulated |
| hsa04062 | Chemokine signaling pathway | 5 | 1.40E-03 | 20 | 9.24 | 0.03 | Upregulated |
| hsa05152 | Tuberculosis | 4 | 0.01 | 20 | 7.77 | 0.18 | Upregulated |
| hsa04610 | Complement and coagulation cascades | 3 | 0.01 | 20 | 14.95 | 0.18 | Upregulated |
| hsa04145 | Phagosome | 3 | 0.06 | 20 | 6.87 | 0.62 | Upregulated |
| hsa04010 | MAPK signaling pathway | 5 | 2.00E-03 | 18 | 7.55 | 0.29 | Downregulated |
| hsa04915 | Estrogen signaling pathway | 3 | 0.02 | 18 | 11.58 | 1 | Downregulated |
| hsa04921 | Oxytocin signaling pathway | 3 | 0.05 | 18 | 7.64 | 1 | Downregulated |
| hsa05200 | Pathways in cancer | 4 | 0.06 | 18 | 3.88 | 1 | Downregulated |

| **ONTOLOGY** | **ID** | **Description** | **Count** | **P. Value** | **List Total** | **Fold Enrichment** | **FDR** |
| --- | --- | --- | --- | --- | --- | --- | --- |
| BP | GO:0098869 | cellular oxidant detoxification | 5 | 3.86E-06 | 27 | 44.42 | 1.50E-03 |
| BP | GO:0002576 | platelet degranulation | 5 | 1.80E-05 | 27 | 30.19 | 3.62E-03 |
| BP | GO:0006954 | inflammatory response | 6 | 2.54E-04 | 27 | 9.84 | 0.03 |
| BP | GO:0051092 | positive regulation of NF-kappaB transcription factor activity | 4 | 1.10E-03 | 27 | 18.70 | 0.11 |
| BP | GO:0032870 | cellular response to hormone stimulus | 3 | 2.19E-03 | 27 | 41.46 | 0.13 |
| CC | GO:0005576 | extracellular region | 11 | 3.99E-05 | 27 | 4.61 | 1.20E-03 |
| CC | GO:0005615 | extracellular space | 10 | 6.32E-05 | 27 | 5.01 | 1.20E-03 |
| CC | GO:0031093 | platelet alpha granule lumen | 4 | 6.44E-05 | 27 | 49.08 | 1.20E-03 |
| CC | GO:0070062 | extracellular exosome | 13 | 2.05E-04 | 27 | 3.12 | 2.86E-03 |
| CC | GO:0072562 | blood microparticle | 4 | 1.28-03 | 27 | 17.76 | 0.01 |
| MF | GO:0016209 | antioxidant activity | 4 | 3.63E-06 | 27 | 125.04 | 3.71E-04 |
| MF | GO:0005543 | phospholipid binding | 3 | 7.70E-03 | 27 | 21.81 | 0.26 |
| MF | GO:0055131 | C3HC4-type RING finger domain binding | 2 | 9.20E-03 | 27 | 208.40 | 0.26 |
| MF | GO:0005178 | integrin binding | 3 | 0.01 | 27 | 17.86 | 0.26 |
| MF | GO:0050786 | RAGE receptor binding | 2 | 0.02 | 27 | 113.67 | 0.26 |

# Supplementary table 5. Top 5 downregulated DEIRGs GO annotation (BP term, CC term, MF term).

BP，Biological process

CC，Cellular component

MF，Molecular function

# Supplementary table 6. Candidate genes in Macrophages.M2-black and Neutrophils-purple modules.

| **Genes** | **GS.Macrophages.M2** | **MMblack** | **Genes** | **GS.Neutrophils** | **MMpurple** |
| --- | --- | --- | --- | --- | --- |
| AASS | 0.525781642 | 0.735072311 | ADGRE3 | 0.723078 | 0.855172 |
| ABCA1 | 0.527666857 | 0.61532621 | ADGRG3 | 0.697134 | 0.666589 |
| ABCA6 | 0.581978707 | 0.656272096 | AQP9 | 0.746601 | 0.860744 |
| ABCC3 | 0.5773286 | 0.779546379 | ATF3 | 0.508185 | 0.596211 |
| ABCC9 | 0.6559285 | 0.63896513 | BTG2 | 0.697924 | 0.779965 |
| ABCE1 | 0.511739581 | 0.616333391 | C5AR1 | 0.578346 | 0.750497 |
| ABHD2 | 0.635549099 | 0.556411775 | CLEC4E | 0.69365 | 0.800852 |
| ACTA2 | 0.560767191 | 0.871074359 | CSF3R | 0.784924 | 0.895047 |
| ACTN1 | 0.64069788 | 0.936386299 | CXCR1 | 0.789257 | 0.884904 |
| ACVR1 | 0.525309163 | 0.646116226 | CXCR2 | 0.872131 | 0.900779 |
| ADAM10 | 0.715375983 | 0.840822973 | DNAJB1 | 0.662331 | 0.567813 |
| ADAM15 | 0.640160946 | 0.500919802 | DUSP1 | 0.777456 | 0.835941 |
| ADAM19 | 0.522636183 | 0.705558174 | EGR1 | 0.562796 | 0.761612 |
| ADAMTS2 | 0.638990477 | 0.733714476 | FCAR | 0.692514 | 0.772881 |
| ADAMTS3 | 0.532580277 | 0.754612875 | FCGR3B | 0.717104 | 0.849724 |
| ADAMTS6 | 0.67204788 | 0.619280374 | FOS | 0.752591 | 0.861875 |
| ADAMTSL3 | 0.607265845 | 0.792859758 | FPR1 | 0.774018 | 0.914355 |
| ADAP2 | 0.535442442 | 0.852851896 | FPR2 | 0.765227 | 0.821219 |
| ADCY3 | 0.589477777 | 0.7493392 | FRAT1 | 0.556865 | 0.627728 |
| ADGRG1 | 0.618299503 | 0.712358672 | HAL | 0.5564 | 0.584566 |
| ADH1A | 0.501472661 | 0.528643628 | HES1 | 0.594908 | 0.720671 |
| ADH1B | 0.586247266 | 0.725842272 | HSPA1A | 0.734052 | 0.722521 |
| AEBP1 | 0.682402881 | 0.922382746 | HSPA1B | 0.740313 | 0.671352 |
| AFF3 | 0.60534146 | 0.586622716 | HSPA8 | 0.545944 | 0.515272 |
| AHNAK2 | 0.624066957 | 0.84431157 | IL1R2 | 0.654671 | 0.744873 |
| AHR | 0.620357153 | 0.824041434 | JUN | 0.781763 | 0.810867 |
| AKAP5 | 0.60638519 | 0.53889345 | JUNB | 0.693427 | 0.860392 |
| ANGPTL2 | 0.658010361 | 0.817804967 | JUND | 0.534681 | 0.655534 |
| ANO1 | 0.692677915 | 0.796849024 | MCL1 | 0.561236 | 0.562347 |
| ANXA3 | 0.567229871 | 0.789116689 | MXD1 | 0.840407 | 0.918426 |
| ANXA4 | 0.511857087 | 0.677585987 | NAMPT | 0.632732 | 0.739314 |
| ANXA5 | 0.599438735 | 0.798521104 | NFIL3 | 0.667994 | 0.714331 |
| APOC1 | 0.68559506 | 0.72395518 | NR4A1 | 0.548007 | 0.637184 |
| ARHGAP11A | 0.666030975 | 0.671436754 | NR4A2 | 0.544029 | 0.60466 |
| ARHGAP15 | 0.504362087 | 0.838705052 | PBX2 | 0.504362 | 0.537052 |
| ARL4C | 0.66757551 | 0.937920396 | PI3 | 0.631535 | 0.674591 |
| ARNTL2 | 0.56872191 | 0.807963325 | PPDPF | 0.544634 | 0.66957 |
| ARSJ | 0.525484421 | 0.698042587 | PTGS2 | 0.693801 | 0.802502 |
| ASPHD1 | 0.514412826 | 0.79564242 | RAD9A | 0.529571 | 0.541324 |
| ASPM | 0.597896513 | 0.678914224 | RGS2 | 0.685767 | 0.790258 |
| ASPN | 0.663872033 | 0.803677988 | S100A12 | 0.752113 | 0.894185 |
| ATP13A3 | 0.649583151 | 0.589819349 | S100A8 | 0.719351 | 0.854107 |
| ATP8B2 | 0.560212197 | 0.734760162 | S100A9 | 0.729618 | 0.905684 |
| AXL | 0.579679573 | 0.667632548 | SELL | 0.560418 | 0.737278 |
| BAZ1A | 0.511508571 | 0.773296648 | SLC11A1 | 0.557221 | 0.683385 |
| BCAT1 | 0.696822822 | 0.763140909 | SOCS3 | 0.551788 | 0.732869 |
| BHLHE41 | 0.68315989 | 0.727684299 | TNFRSF10C | 0.514468 | 0.653484 |
| BICC1 | 0.575242559 | 0.696574868 | TREM1 | 0.820249 | 0.881845 |
| BIRC5 | 0.580477094 | 0.639406826 | VNN2 | 0.771441 | 0.902632 |
| BMP5 | 0.565785667 | 0.542832374 | ZFP36 | 0.72624 | 0.889825 |
| BRCA2 | 0.651078427 | 0.587944422 | ZNF331 | 0.583118 | 0.555365 |
| BUB1B | 0.5267778 | 0.801668688 |  |  |  |
| C11orf80 | 0.580203498 | 0.729504533 |  |  |  |
| C1orf54 | 0.526085156 | 0.638474753 |  |  |  |
| C2CD2 | 0.50746977 | 0.591491047 |  |  |  |
| C3 | 0.63540442 | 0.877853468 |  |  |  |
| C3AR1 | 0.548901551 | 0.838856331 |  |  |  |
| C7 | 0.587825938 | 0.676723045 |  |  |  |
| CABYR | 0.551655474 | 0.646723182 |  |  |  |
| CAD | 0.500303274 | 0.635334469 |  |  |  |
| CADM3 | 0.525568006 | 0.61619217 |  |  |  |
| CAMSAP1 | 0.583461684 | 0.562512474 |  |  |  |
| CASKIN2 | 0.522910241 | 0.532703977 |  |  |  |
| CASP3 | 0.601805992 | 0.645856629 |  |  |  |
| CAV1 | 0.637619165 | 0.808675552 |  |  |  |
| CBFB | 0.639174609 | 0.734367689 |  |  |  |
| CCL11 | 0.608722847 | 0.747709986 |  |  |  |
| CCL18 | 0.635592108 | 0.667760589 |  |  |  |
| CCL2 | 0.59972881 | 0.845762985 |  |  |  |
| CCL21 | 0.638209104 | 0.760143895 |  |  |  |
| CCL8 | 0.516647094 | 0.720808057 |  |  |  |
| CCNA2 | 0.640540358 | 0.678606846 |  |  |  |
| CCNB1 | 0.509264964 | 0.686588816 |  |  |  |
| CCND2 | 0.679885137 | 0.790082056 |  |  |  |
| CD163 | 0.550604419 | 0.790760655 |  |  |  |
| CD180 | 0.50743245 | 0.635597993 |  |  |  |
| CD1C | 0.555870334 | 0.743257589 |  |  |  |
| CD209 | 0.713814949 | 0.700694115 |  |  |  |
| CD24 | 0.612252216 | 0.735280967 |  |  |  |
| CD248 | 0.63761598 | 0.811419183 |  |  |  |
| CD33 | 0.521064064 | 0.716370274 |  |  |  |
| CD44 | 0.61322962 | 0.92965722 |  |  |  |
| CD68 | 0.571474074 | 0.734821995 |  |  |  |
| CD70 | 0.535832551 | 0.588317939 |  |  |  |
| CD84 | 0.574655454 | 0.682785078 |  |  |  |
| CDH11 | 0.596636199 | 0.846218441 |  |  |  |
| CDH2 | 0.524628856 | 0.653062676 |  |  |  |
| CDH6 | 0.733428425 | 0.761208813 |  |  |  |
| CDKN2B | 0.531462888 | 0.730473932 |  |  |  |
| CDKN3 | 0.564662655 | 0.763453925 |  |  |  |
| CENPF | 0.704767115 | 0.790742335 |  |  |  |
| CENPU | 0.515710716 | 0.743822468 |  |  |  |
| CFH | 0.609556322 | 0.780352769 |  |  |  |
| CH25H | 0.568034819 | 0.773555555 |  |  |  |
| CHST15 | 0.507240958 | 0.866967924 |  |  |  |
| CLCF1 | 0.539936735 | 0.576353829 |  |  |  |
| CLDN1 | 0.659483081 | 0.767755737 |  |  |  |
| CLEC10A | 0.566175638 | 0.886565519 |  |  |  |
| CLEC2D | 0.574195265 | 0.644862774 |  |  |  |
| CLEC5A | 0.51720114 | 0.756884573 |  |  |  |
| CLU | 0.552174734 | 0.841978933 |  |  |  |
| CNTNAP1 | 0.543930417 | 0.781504164 |  |  |  |
| COL10A1 | 0.712022003 | 0.7148273 |  |  |  |
| COL11A1 | 0.558518604 | 0.796045056 |  |  |  |
| COL14A1 | 0.568194135 | 0.662567819 |  |  |  |
| COL15A1 | 0.638393986 | 0.880559662 |  |  |  |
| COL1A1 | 0.623062994 | 0.919967893 |  |  |  |
| COL1A2 | 0.644509532 | 0.920703567 |  |  |  |
| COL3A1 | 0.613039898 | 0.847826465 |  |  |  |
| COL4A1 | 0.631314732 | 0.783588404 |  |  |  |
| COL4A2 | 0.615892203 | 0.787393282 |  |  |  |
| COL6A1 | 0.630795511 | 0.743870257 |  |  |  |
| COL6A2 | 0.611755052 | 0.912805495 |  |  |  |
| COL6A3 | 0.590827602 | 0.908358509 |  |  |  |
| CORO1C | 0.622121463 | 0.886851761 |  |  |  |
| CP | 0.621253928 | 0.738894753 |  |  |  |
| CPA3 | 0.517400883 | 0.804184384 |  |  |  |
| CPE | 0.510419917 | 0.840200398 |  |  |  |
| CPVL | 0.547488793 | 0.846809637 |  |  |  |
| CREB5 | 0.621517615 | 0.716969165 |  |  |  |
| CRIP1 | 0.55835677 | 0.827450871 |  |  |  |
| CRISPLD2 | 0.500775246 | 0.551059043 |  |  |  |
| CSF2RA | 0.510090713 | 0.744527659 |  |  |  |
| CTSC | 0.661230627 | 0.798829171 |  |  |  |
| CTSK | 0.593659437 | 0.770141916 |  |  |  |
| CTSV | 0.596308837 | 0.809037289 |  |  |  |
| CYBRD1 | 0.548662658 | 0.720349643 |  |  |  |
| CYTL1 | 0.568383628 | 0.700792297 |  |  |  |
| DACT1 | 0.592920382 | 0.884285715 |  |  |  |
| DDR2 | 0.667111918 | 0.644347336 |  |  |  |
| DIAPH1 | 0.54228574 | 0.6543597 |  |  |  |
| DIAPH3 | 0.564095908 | 0.594585467 |  |  |  |
| DLGAP5 | 0.547109644 | 0.82534621 |  |  |  |
| DNMT3B | 0.540568614 | 0.656290006 |  |  |  |
| DOCK10 | 0.516874793 | 0.832889388 |  |  |  |
| DONSON | 0.525029361 | 0.643541876 |  |  |  |
| DSP | 0.591016099 | 0.694409654 |  |  |  |
| DTL | 0.607745128 | 0.724640104 |  |  |  |
| ECT2 | 0.547360532 | 0.800294619 |  |  |  |
| EDNRA | 0.674549708 | 0.617940151 |  |  |  |
| EFNA5 | 0.576201427 | 0.645496404 |  |  |  |
| ELN | 0.607216389 | 0.780322187 |  |  |  |
| ENAH | 0.772164045 | 0.866536695 |  |  |  |
| ENPP1 | 0.551108723 | 0.58911654 |  |  |  |
| ENTPD1 | 0.559872429 | 0.882129681 |  |  |  |
| ENTPD7 | 0.506973077 | 0.682884419 |  |  |  |
| EPB41L2 | 0.630661346 | 0.770096645 |  |  |  |
| EPHA3 | 0.557130796 | 0.640794156 |  |  |  |
| EPHB2 | 0.658958538 | 0.707847533 |  |  |  |
| ESM1 | 0.52256366 | 0.693096256 |  |  |  |
| ETV1 | 0.650328595 | 0.697874971 |  |  |  |
| ETV4 | 0.568937685 | 0.571732736 |  |  |  |
| ETV5 | 0.605509448 | 0.667874521 |  |  |  |
| EXT1 | 0.673359581 | 0.846901863 |  |  |  |
| FAM110B | 0.568397509 | 0.818353738 |  |  |  |
| FAM155A | 0.562915941 | 0.654011194 |  |  |  |
| FAP | 0.622083925 | 0.857804158 |  |  |  |
| FBLN5 | 0.661307705 | 0.713091455 |  |  |  |
| FBN1 | 0.708722439 | 0.84766198 |  |  |  |
| FCER1A | 0.509668327 | 0.773235341 |  |  |  |
| FGF2 | 0.505253028 | 0.533863574 |  |  |  |
| FILIP1L | 0.670672929 | 0.843132666 |  |  |  |
| FKBP10 | 0.677636964 | 0.698363291 |  |  |  |
| FLNA | 0.516257831 | 0.71067601 |  |  |  |
| FLNB | 0.707209397 | 0.763709847 |  |  |  |
| FMO3 | 0.59345899 | 0.798135522 |  |  |  |
| FMOD | 0.579627786 | 0.837747405 |  |  |  |
| FN1 | 0.786302179 | 0.798554459 |  |  |  |
| FOLR2 | 0.640764852 | 0.841801447 |  |  |  |
| FPR3 | 0.75229228 | 0.842581707 |  |  |  |
| FUT8 | 0.542097445 | 0.591364514 |  |  |  |
| GADD45B | 0.5929868 | 0.669879122 |  |  |  |
| GAL3ST4 | 0.537011251 | 0.787696161 |  |  |  |
| GALNT1 | 0.595514141 | 0.794435191 |  |  |  |
| GALNT6 | 0.521129201 | 0.739684311 |  |  |  |
| GFRA1 | 0.697747655 | 0.758338424 |  |  |  |
| GGT5 | 0.56933276 | 0.848420429 |  |  |  |
| GLI1 | 0.585938353 | 0.709374856 |  |  |  |
| GLI2 | 0.655856084 | 0.696084297 |  |  |  |
| GLIPR1 | 0.552607467 | 0.890282323 |  |  |  |
| GPHN | 0.6103756 | 0.770189324 |  |  |  |
| GPR176 | 0.672565822 | 0.741246727 |  |  |  |
| GPR183 | 0.516776474 | 0.720005627 |  |  |  |
| GPR39 | 0.511764192 | 0.625705662 |  |  |  |
| GPSM2 | 0.54163094 | 0.718648485 |  |  |  |
| GTSE1 | 0.556189852 | 0.607437706 |  |  |  |
| HDAC9 | 0.596443474 | 0.703241551 |  |  |  |
| HEYL | 0.664114187 | 0.671878902 |  |  |  |
| HGF | 0.637737395 | 0.694010781 |  |  |  |
| HIF1A | 0.58113841 | 0.735254735 |  |  |  |
| HMMR | 0.55908569 | 0.80201585 |  |  |  |
| HOMER3 | 0.551535092 | 0.799267073 |  |  |  |
| HOXA11 | 0.63293915 | 0.733138681 |  |  |  |
| HOXD10 | 0.523237048 | 0.552680478 |  |  |  |
| HOXD11 | 0.529695205 | 0.73179166 |  |  |  |
| HRH1 | 0.590662779 | 0.788405383 |  |  |  |
| HS3ST1 | 0.513163589 | 0.816402849 |  |  |  |
| HSPB6 | 0.558800484 | 0.577115501 |  |  |  |
| HSPG2 | 0.621808275 | 0.690613373 |  |  |  |
| IGFBP1 | 0.502774096 | 0.642913891 |  |  |  |
| IGFBP3 | 0.602368262 | 0.810298019 |  |  |  |
| IGFBP6 | 0.670134205 | 0.904283858 |  |  |  |
| IL18 | 0.684140028 | 0.7350079 |  |  |  |
| IL32 | 0.56403848 | 0.76246808 |  |  |  |
| IL33 | 0.537504164 | 0.818690539 |  |  |  |
| INHBA | 0.689399942 | 0.76298125 |  |  |  |
| IRF8 | 0.500500171 | 0.902987961 |  |  |  |
| ISLR | 0.609302682 | 0.880083174 |  |  |  |
| ITGA8 | 0.535409136 | 0.533911553 |  |  |  |
| ITGB6 | 0.601126568 | 0.767901908 |  |  |  |
| ITPR3 | 0.661988535 | 0.801326235 |  |  |  |
| JAG1 | 0.671992684 | 0.694939919 |  |  |  |
| KCNJ8 | 0.507442979 | 0.812886077 |  |  |  |
| KCTD12 | 0.505422942 | 0.633844061 |  |  |  |
| KDM5B | 0.577953748 | 0.67345716 |  |  |  |
| KIAA0895 | 0.562575492 | 0.624890955 |  |  |  |
| KIF11 | 0.513242964 | 0.62726546 |  |  |  |
| KIF23 | 0.567626584 | 0.692199408 |  |  |  |
| KLF15 | 0.522420518 | 0.586564199 |  |  |  |
| KLF5 | 0.523758854 | 0.608068938 |  |  |  |
| KLHL4 | 0.632127827 | 0.71714589 |  |  |  |
| KNTC1 | 0.594837405 | 0.558911302 |  |  |  |
| KPNA2 | 0.513550411 | 0.672680948 |  |  |  |
| KRT17 | 0.512811067 | 0.697389916 |  |  |  |
| KRT19 | 0.59033278 | 0.823363785 |  |  |  |
| LAIR1 | 0.522404397 | 0.846742772 |  |  |  |
| LAMA2 | 0.541563236 | 0.772760317 |  |  |  |
| LAMA4 | 0.731882418 | 0.747838463 |  |  |  |
| LAMB1 | 0.59567424 | 0.726502335 |  |  |  |
| LAMC1 | 0.614958218 | 0.783461876 |  |  |  |
| LAMC2 | 0.655427896 | 0.776114172 |  |  |  |
| LAMP5 | 0.587405349 | 0.608878828 |  |  |  |
| LEF1 | 0.594194446 | 0.683140835 |  |  |  |
| LGALS3 | 0.608584862 | 0.786421942 |  |  |  |
| LGALS9 | 0.537100625 | 0.538450615 |  |  |  |
| LMCD1 | 0.53089025 | 0.605690861 |  |  |  |
| LOXL2 | 0.5356871 | 0.797279167 |  |  |  |
| LPAR1 | 0.648604021 | 0.855016082 |  |  |  |
| LRP1 | 0.617660563 | 0.735507731 |  |  |  |
| LTBP1 | 0.687499516 | 0.691291265 |  |  |  |
| LTBP2 | 0.591023647 | 0.764996649 |  |  |  |
| LUM | 0.698989291 | 0.820711012 |  |  |  |
| LY86 | 0.56074631 | 0.786337226 |  |  |  |
| LYPD1 | 0.591977335 | 0.869774175 |  |  |  |
| LYVE1 | 0.704869111 | 0.711322494 |  |  |  |
| MAN2B1 | 0.511901994 | 0.896232665 |  |  |  |
| MAP3K1 | 0.689133532 | 0.812069013 |  |  |  |
| MAP4K4 | 0.62904513 | 0.758604161 |  |  |  |
| MAP7D3 | 0.538051638 | 0.630556983 |  |  |  |
| MARCKS | 0.737581447 | 0.869121536 |  |  |  |
| MATN2 | 0.602197754 | 0.74336206 |  |  |  |
| MCAM | 0.614289523 | 0.81845698 |  |  |  |
| MCTP1 | 0.617117254 | 0.848234726 |  |  |  |
| MDK | 0.612169871 | 0.862358822 |  |  |  |
| MECOM | 0.525521721 | 0.603614196 |  |  |  |
| MEST | 0.541715813 | 0.82422787 |  |  |  |
| MET | 0.561758406 | 0.670395483 |  |  |  |
| MFAP2 | 0.591501965 | 0.764169504 |  |  |  |
| MFAP4 | 0.62463635 | 0.756982205 |  |  |  |
| MFGE8 | 0.532853611 | 0.791012658 |  |  |  |
| MICAL2 | 0.61405299 | 0.662839114 |  |  |  |
| MILR1 | 0.646419624 | 0.836836405 |  |  |  |
| MKI67 | 0.659449623 | 0.677545738 |  |  |  |
| MLLT11 | 0.568124359 | 0.871528571 |  |  |  |
| MMP11 | 0.548813145 | 0.723259929 |  |  |  |
| MMP14 | 0.637928569 | 0.602592716 |  |  |  |
| MMP19 | 0.509026762 | 0.593593109 |  |  |  |
| MMP2 | 0.587398947 | 0.90354912 |  |  |  |
| MMP7 | 0.71200526 | 0.798938391 |  |  |  |
| MOXD1 | 0.705594074 | 0.897013612 |  |  |  |
| MRAS | 0.534419267 | 0.785650511 |  |  |  |
| MRC1 | 0.545431882 | 0.730900123 |  |  |  |
| MS4A4A | 0.513781542 | 0.841286996 |  |  |  |
| MS4A6A | 0.671477047 | 0.916424629 |  |  |  |
| MSC | 0.566447647 | 0.728404759 |  |  |  |
| MSR1 | 0.741209115 | 0.707220866 |  |  |  |
| MYH10 | 0.634679039 | 0.723620643 |  |  |  |
| MYOF | 0.659678548 | 0.729958054 |  |  |  |
| NCAPG | 0.575472021 | 0.755705055 |  |  |  |
| NCKAP1L | 0.566138491 | 0.744145451 |  |  |  |
| NDC80 | 0.572777881 | 0.830579222 |  |  |  |
| NEFL | 0.651094705 | 0.818418931 |  |  |  |
| NEFM | 0.591888715 | 0.786252415 |  |  |  |
| NFIX | 0.632310985 | 0.669312931 |  |  |  |
| NID2 | 0.651051755 | 0.758148122 |  |  |  |
| NLGN4X | 0.600472453 | 0.826734952 |  |  |  |
| NOTCH3 | 0.561840684 | 0.742755257 |  |  |  |
| NPM1 | 0.588816409 | 0.695000895 |  |  |  |
| NR2F2 | 0.536934471 | 0.693031702 |  |  |  |
| NRP2 | 0.691512528 | 0.607857254 |  |  |  |
| NT5DC3 | 0.64528219 | 0.731527789 |  |  |  |
| NT5E | 0.549922851 | 0.600538418 |  |  |  |
| NTRK3 | 0.560110212 | 0.544863564 |  |  |  |
| NUAK1 | 0.535800335 | 0.640389619 |  |  |  |
| NUP160 | 0.513534538 | 0.551558211 |  |  |  |
| NUP93 | 0.55088079 | 0.69109092 |  |  |  |
| NUSAP1 | 0.609270598 | 0.739289088 |  |  |  |
| OLFML2B | 0.720617317 | 0.873178713 |  |  |  |
| OSBPL3 | 0.599035536 | 0.822180584 |  |  |  |
| OSMR | 0.625161025 | 0.77018479 |  |  |  |
| OXSR1 | 0.51247193 | 0.588845939 |  |  |  |
| P2RY14 | 0.54638744 | 0.810090579 |  |  |  |
| PALMD | 0.618918678 | 0.601332024 |  |  |  |
| PAX8 | 0.51591532 | 0.567923327 |  |  |  |
| PBK | 0.532004262 | 0.774437626 |  |  |  |
| PCDH17 | 0.52566437 | 0.610820816 |  |  |  |
| PCNX2 | 0.56284522 | 0.670616002 |  |  |  |
| PCOLCE | 0.552230449 | 0.896415732 |  |  |  |
| PCSK7 | 0.645419836 | 0.652485591 |  |  |  |
| PDE1A | 0.608051294 | 0.760184736 |  |  |  |
| PDE3A | 0.506958884 | 0.540643593 |  |  |  |
| PDE5A | 0.642329863 | 0.643076758 |  |  |  |
| PDGFB | 0.550902143 | 0.544620618 |  |  |  |
| PDGFC | 0.510518229 | 0.568857273 |  |  |  |
| PDK4 | 0.61845096 | 0.556110512 |  |  |  |
| PDLIM1 | 0.504197439 | 0.735965594 |  |  |  |
| PECAM1 | 0.501963815 | 0.553298208 |  |  |  |
| PHLDA3 | 0.537453921 | 0.638051824 |  |  |  |
| PID1 | 0.56624156 | 0.829222803 |  |  |  |
| PKD2 | 0.562181103 | 0.692354204 |  |  |  |
| PLA2G7 | 0.533331369 | 0.802414046 |  |  |  |
| PLAU | 0.558803969 | 0.842795998 |  |  |  |
| PLCB4 | 0.523581402 | 0.597182444 |  |  |  |
| PLK2 | 0.623311804 | 0.783629612 |  |  |  |
| PLN | 0.54952024 | 0.630085091 |  |  |  |
| PLXDC2 | 0.515211701 | 0.537949607 |  |  |  |
| PMP22 | 0.583655087 | 0.866554473 |  |  |  |
| POSTN | 0.540284076 | 0.716954394 |  |  |  |
| PPP1R3C | 0.573374267 | 0.756991674 |  |  |  |
| PRCP | 0.514297841 | 0.593033264 |  |  |  |
| PRELP | 0.664619021 | 0.776377032 |  |  |  |
| PROCR | 0.503309457 | 0.699489599 |  |  |  |
| PROM1 | 0.638517858 | 0.764102264 |  |  |  |
| PRRX1 | 0.521583734 | 0.745806731 |  |  |  |
| PSME4 | 0.685046706 | 0.726731227 |  |  |  |
| PTEN | 0.61763134 | 0.833330559 |  |  |  |
| PTGES | 0.618813384 | 0.657905501 |  |  |  |
| PTGS1 | 0.561330688 | 0.628349917 |  |  |  |
| PTN | 0.6092975 | 0.764231214 |  |  |  |
| PTPRK | 0.570672328 | 0.700757103 |  |  |  |
| PTTG1 | 0.585379242 | 0.780713368 |  |  |  |
| PUS7 | 0.575262058 | 0.658763825 |  |  |  |
| PXDN | 0.649416238 | 0.889836867 |  |  |  |
| RAB32 | 0.629779195 | 0.803762831 |  |  |  |
| RAB3B | 0.617628942 | 0.62342031 |  |  |  |
| RAD51AP1 | 0.512530456 | 0.604082428 |  |  |  |
| RASSF9 | 0.727661723 | 0.571587199 |  |  |  |
| RBP1 | 0.5972135 | 0.765817339 |  |  |  |
| RCC1 | 0.515476269 | 0.681690169 |  |  |  |
| RELN | 0.60769168 | 0.668856095 |  |  |  |
| REV3L | 0.556690286 | 0.55224207 |  |  |  |
| RFTN1 | 0.535950207 | 0.820598495 |  |  |  |
| RGS4 | 0.672599819 | 0.899210874 |  |  |  |
| RGS5 | 0.641331987 | 0.79812529 |  |  |  |
| RNASE6 | 0.609112732 | 0.85362495 |  |  |  |
| RNF125 | 0.505157733 | 0.660861278 |  |  |  |
| ROR1 | 0.543873494 | 0.683204781 |  |  |  |
| S100A3 | 0.587813228 | 0.758213316 |  |  |  |
| SACS | 0.60322195 | 0.869333327 |  |  |  |
| SAMHD1 | 0.534171479 | 0.701762506 |  |  |  |
| SCARA3 | 0.54048571 | 0.914942906 |  |  |  |
| SDS | 0.507379211 | 0.614828943 |  |  |  |
| SEC23A | 0.512818429 | 0.537004737 |  |  |  |
| SEC24D | 0.50906548 | 0.776709923 |  |  |  |
| SEL1L3 | 0.575838055 | 0.834961502 |  |  |  |
| SERBP1 | 0.535574129 | 0.579687626 |  |  |  |
| SERPINE1 | 0.500568613 | 0.580577284 |  |  |  |
| SERPINE2 | 0.52226937 | 0.727309868 |  |  |  |
| SERPINF1 | 0.553414762 | 0.810863161 |  |  |  |
| SETBP1 | 0.630214092 | 0.647435233 |  |  |  |
| SGCD | 0.531718405 | 0.572106538 |  |  |  |
| SLAMF1 | 0.512437588 | 0.612099573 |  |  |  |
| SLC12A2 | 0.734035439 | 0.680060615 |  |  |  |
| SLC22A3 | 0.514528257 | 0.699460502 |  |  |  |
| SLC34A2 | 0.504143143 | 0.598121427 |  |  |  |
| SLC38A1 | 0.575124218 | 0.768762143 |  |  |  |
| SLC6A4 | 0.505142783 | 0.747643695 |  |  |  |
| SLC6A6 | 0.71493008 | 0.565164994 |  |  |  |
| SLC7A2 | 0.55412212 | 0.642298608 |  |  |  |
| SLCO2B1 | 0.585312841 | 0.698934136 |  |  |  |
| SLIT3 | 0.712714912 | 0.74695196 |  |  |  |
| SORBS2 | 0.57207847 | 0.593132461 |  |  |  |
| SOWAHC | 0.518116236 | 0.614483266 |  |  |  |
| SOX4 | 0.677805863 | 0.815807223 |  |  |  |
| SPATS2 | 0.619189246 | 0.778940053 |  |  |  |
| STK17A | 0.528260283 | 0.818567905 |  |  |  |
| STK38L | 0.555787343 | 0.729769759 |  |  |  |
| STK39 | 0.506617799 | 0.698993297 |  |  |  |
| SVEP1 | 0.606456564 | 0.623790495 |  |  |  |
| SVIL | 0.590234577 | 0.63479261 |  |  |  |
| SYK | 0.535399441 | 0.770759965 |  |  |  |
| SYT11 | 0.675164798 | 0.796797435 |  |  |  |
| SYTL2 | 0.749995071 | 0.802823106 |  |  |  |
| TAGLN | 0.574370218 | 0.896921637 |  |  |  |
| TBC1D16 | 0.684228015 | 0.715917423 |  |  |  |
| TBC1D4 | 0.550662338 | 0.638299036 |  |  |  |
| TES | 0.570483116 | 0.83814061 |  |  |  |
| TGFB2 | 0.746210642 | 0.728118965 |  |  |  |
| TGFB3 | 0.632697618 | 0.721342214 |  |  |  |
| TGFBI | 0.583598793 | 0.898840021 |  |  |  |
| TGFBR1 | 0.630913178 | 0.601689641 |  |  |  |
| THBD | 0.566097809 | 0.768576133 |  |  |  |
| THBS2 | 0.586020675 | 0.929095662 |  |  |  |
| THSD4 | 0.517688311 | 0.598177956 |  |  |  |
| TLR7 | 0.598804055 | 0.809229939 |  |  |  |
| TM6SF1 | 0.717758817 | 0.800768676 |  |  |  |
| TNC | 0.629624874 | 0.853558202 |  |  |  |
| TNFRSF10D | 0.517165651 | 0.595354096 |  |  |  |
| TNFRSF11B | 0.582010815 | 0.680324174 |  |  |  |
| TNFRSF12A | 0.560452959 | 0.790218703 |  |  |  |
| TNXB | 0.724726436 | 0.626149183 |  |  |  |
| TOP2A | 0.650421826 | 0.77877925 |  |  |  |
| TP53 | 0.60560487 | 0.82175684 |  |  |  |
| TP53I11 | 0.577449378 | 0.536249858 |  |  |  |
| TPM1 | 0.747440037 | 0.832303992 |  |  |  |
| TPM4 | 0.555485815 | 0.771303219 |  |  |  |
| TPX2 | 0.628841502 | 0.748770602 |  |  |  |
| TREM2 | 0.619554558 | 0.676271014 |  |  |  |
| TRIM27 | 0.520415692 | 0.652122888 |  |  |  |
| TRIO | 0.805437417 | 0.685548747 |  |  |  |
| TRO | 0.58721888 | 0.743735774 |  |  |  |
| TSHZ2 | 0.641623269 | 0.682937725 |  |  |  |
| TTC3 | 0.578834373 | 0.58970525 |  |  |  |
| TUBB2B | 0.528261547 | 0.758210656 |  |  |  |
| TYMS | 0.589416827 | 0.73509297 |  |  |  |
| UBE2C | 0.600207519 | 0.756394663 |  |  |  |
| VASH1 | 0.644127754 | 0.748885838 |  |  |  |
| VCAM1 | 0.592268646 | 0.743297758 |  |  |  |
| VCAN | 0.690945985 | 0.856013115 |  |  |  |
| VEGFC | 0.648132796 | 0.75171976 |  |  |  |
| VSIG4 | 0.691625562 | 0.902823456 |  |  |  |
| WNT2B | 0.57352997 | 0.754411566 |  |  |  |
| ZFHX4 | 0.544332605 | 0.665079793 |  |  |  |
| ZSCAN16 | 0.537537734 | 0.606066087 |  |  |  |
| ZWILCH | 0.544220278 | 0.609831452 |  |  |  |

GS, Gene significance

MM, module membership

# Supplementary table 7. miRNAs prediction from Mienturnet

| **microRNA** | **p-value** | **Target Gene 1** | **Target Gene 2** | **Species** | **Organ** |
| --- | --- | --- | --- | --- | --- |
| hsa-miR-1273g-3p | 0.003 | VEGFC | C3 | homo sapiens | Kidney |
| hsa-miR-4252 | 0.002 | C3 | VEGFC | homo sapiens | Kidney |
| hsa-miR-4793-3p | 0.002 | VEGFC | C3 | homo sapiens | Kidney |
| hsa-miR-6512-3p | 0.003 | C3 | VEGFC | homo sapiens | Kidney |
| hsa-miR-661 | 0.002 | C3 | VEGFC | homo sapiens | Kidney |
| hsa-miR-6720-5p | 0.003 | C3 | VEGFC | homo sapiens | Kidney |
| hsa-miR-7703 | 0.002 | VEGFC | C3 | homo sapiens | Kidney |
| hsa-miR-939-3p | 0.002 | C3 | VEGFC | homo sapiens | Kidney |
| hsa-miR-122-5p | 0.004 | C3 | VEGFC | homo sapiens | Kidney |
| hsa-miR-766-3p | 0.004 | C3 | VEGFC | homo sapiens | Kidney |
| hsa-miR-6849-3p | 0.006 | C3 | VEGFC | homo sapiens | Kidney |
| hsa-miR-218-5p | 0.008 | VEGFC | FN1 | homo sapiens | Kidney |
| hsa-miR-26b-5p | 0.042 | FN1 | C3 | homo sapiens | Kidney |

# Supplementary table 8. The correlation between immune-related biomarkers and immune cells.

| **FN1** | **cor** | **p. value** | **C3** | **cor** | **p. value** | **VEGFC** | **cor** | **p. value** |
| --- | --- | --- | --- | --- | --- | --- | --- | --- |
| B cells naive | -0.02 | 0.85 | B cells naive | -0.01 | 0.88 | B cells naive | -0.18 | 0.06 |
| B cells memory | 0.05 | 0.63 | B cells memory | 0.03 | 0.74 | B cells memory | 0.07 | 0.45 |
| Plasma cells | 0.07 | 0.47 | Plasma cells | 0.17 | 0.07 | Plasma cells | 0.00 | 0.97 |
| T cells CD8 | -0.08 | 0.43 | T cells CD8 | -0.11 | 0.26 | T cells CD8 | -0.21 | 0.03 |
| T cells CD4 memory resting | -0.25 | 0.01 | T cells CD4 memory resting | -0.30 | 0.002 | T cells CD4 memory resting | -0.22 | 0.02 |
| T cells CD4 memory activated | 0.21 | 0.03 | T cells CD4 memory activated | 0.16 | 0.11 | T cells CD4 memory activated | 0.00 | 0.99 |
| T cells regulatory (Tregs) | -0.21 | 0.03 | T cells regulatory (Tregs) | -0.28 | 0.003 | T cells regulatory (Tregs) | -0.19 | 0.05 |
| T cells gamma delta | 0.30 | 2.61E-03 | T cells gamma delta | 0.48 | 4.26E-07 | T cells gamma delta | 0.33 | 0.0007 |
| NK cells resting | -0.31 | 1.74E-03 | NK cells resting | -0.28 | 0.003 | NK cells resting | -0.27 | 0.006 |
| NK cells activated | -0.03 | 0.77 | NK cells activated | -0.11 | 0.28 | NK cells activated | 0.13 | 0.18 |
| Monocytes | -0.17 | 0.08 | Monocytes | -0.07 | 0.47 | Monocytes | 0.08 | 0.44 |
| Macrophages M1 | 0.14 | 0.16 | Macrophages M1 | -0.04 | 0.66 | Macrophages M1 | 0.20 | 0.04 |
| Macrophages M2 | 0.79 | 3.00E-23 | Macrophages M2 | 0.65 | 2.00E-13 | Macrophages M2 | 0.64 | 5.37E-13 |
| Dendritic cells resting | 0.04 | 0.71 | Dendritic cells resting | -0.14 | 0.15 | Dendritic cells resting | -0.07 | 0.48 |
| Dendritic cells activated | 0.24 | 0.01 | Dendritic cells activated | 0.38 | 8.38E-05 | Dendritic cells activated | 0.11 | 0.25 |
| Mast cells resting | 0.42 | 1.34E-05 | Mast cells resting | 0.40 | 3.04E-05 | Mast cells resting | 0.23 | 0.02 |
| Mast cells activated | -0.43 | 7.94E-06 | Mast cells activated | -0.36 | 0.0001 | Mast cells activated | -0.38 | 8.42E-05 |
| Neutrophils | -0.44 | 6.50E-09 | Neutrophils | -0.40 | 3.13E-05 | Neutrophils | -0.45 | 1.79E-06 |
